# Sonogenetic Control of Engineered Probiotics for the Treatment of MASH by Modulating the Gut‐Liver Axis

**DOI:** 10.1002/advs.78100

**Published:** 2026-10-08

**Authors:** Chunya Ji, Yan Fang, Shen Zhang, Weichen Xu, Wenyun Guo, Jing Chen, Mingrui Zhu, Congjian Wen, Siming Chen, Yuli Zhu, Huixiong Xu, Haohao Yin

**Affiliations:** ^1^ Department of Ultrasound Institute of Ultrasound in Medicine and Engineering Zhongshan Hospital Fudan University Shanghai China; ^2^ Department of Medical Ultrasound Center of Minimally Invasive Treatment for Tumor School of Medicine Shanghai Tenth People's Hospital Tongji University Shanghai China; ^3^ Shanghai Engineering Research Center of Ultrasound Diagnosis and Treatment Shanghai China; ^4^ School of Data Science Fudan University Shanghai China

**Keywords:** engineered probiotics, gut‐liver axis, interleukin‐22, MASH, sonogenetics

## Abstract

Metabolic dysfunction‐associated steatohepatitis (MASH) currently lacks effective targeted therapeutics, while interleukin‐22 (IL‐22) as a potential treatment approach faces challenges in the clinical translation due to off‐target risks associated with systemic delivery. To address this limitation, we developed an intelligent oral biologics platform centered on an engineered probiotic strain (*Escherichia coli* Nissle 1917) capable of producing therapeutic IL‐22 in situ, encapsulated within an inulin hydrogel to resist gastric degradation. Critically, we integrated a sonogenetic control module that enables non‐invasive, spatiotemporally precise activation of IL‐22 expression in the gut via remote ultrasound stimulation. In MASH model, this targeted, on‐demand IL‐22 delivery effectively remodeled the gut microbiota and improved intestinal barrier function. This led to significantly reduced portal lipopolysaccharide (LPS) levels, which in turn decreased hepatic IL‐6 production. Mechanistically, this strategy inhibited the pathological overactivation of the PI3K‐AKT‐mTOR signaling pathway, thereby markedly ameliorating hepatic steatosis, inflammation, and fibrosis. Notably, by confining IL‐22 production to the gut, this strategy completely circumvented the off‐target effects of conventional IL‐22 therapy. We observed reversal of MASH‐associated cognitive impairment, highlighting systemic benefits of restoring gut‐liver axis homeostasis. This study establishes a transformative, multi‐beneficial MASH therapy paradigm by modulating the gut‐liver axis, offering a safe, effective, clinically translatable strategy.

## Graphical abstract

Researchers developed an engineered probiotic encapsulated in an inulin hydrogel, enabling ultrasound‐triggered intestinal delivery of IL‐22. In MASH model mice, this system remodeled gut microbiota, strengthened the intestinal barrier and reduced portal LPS and hepatic IL‐6. By suppressing overactive PI3K‐AKT‐mTOR signaling, it significantly alleviated both MASH and associated cognitive impairment.

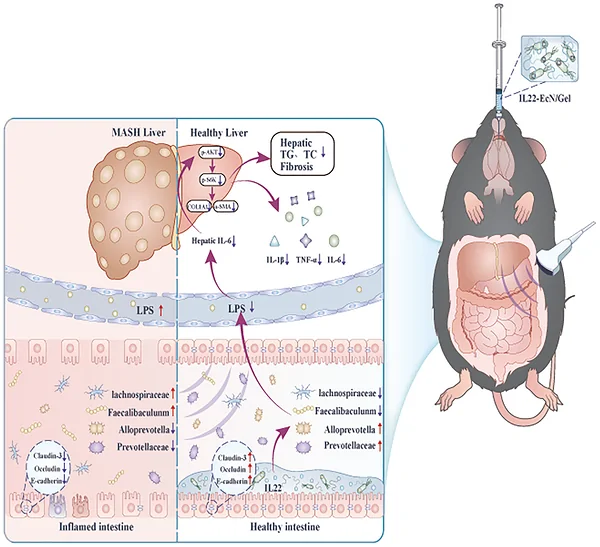

## 1 Introduction

Metabolic dysfunction‐associated steatotic liver disease (MASLD) now represents the most prevalent chronic liver disease worldwide, with its incidence steadily increasing to approximately 38% [1, 2]. As a progressive stage of MASLD, metabolic dysfunction‐associated steatohepatitis (MASH) is distinguished not only by hepatic steatosis, but also by significant inflammation and hepatocyte damage. Furthermore, it is clear that MASLD/MASH has been closely associated with cognitive impairment and cerebral dysfunction [3, 4, 5]. These complications substantially amplify the overall disease burden and present severe challenges for the development of clinical treatment strategies. MASLD is reversible before progressing to cirrhosis, which makes MASH a critical therapeutic target for pharmacological intervention [6]. How to treat MASH safely and effectively has always been a global research priority. The recent U.S. Food and Drug Administration (FDA) approval of Resmetirom (a selective thyroid hormone receptor‐beta [THR‐β] agonist) and Semaglutide (a glucagon‐like peptide‐1 [GLP‐1] receptor agonist) marked a historic milestone, as they became the first drugs for MASH patients with moderate to advanced liver fibrosis (stage F2‐F3) [7, 8]. However, practice guidance of the American Association for the Study of Liver Diseases (AASLD) and relevant phase 3 clinical trials indicated that these two drugs were not suitable for all MASH patients. In addition, long‐term use may induce adverse effects, notably gastrointestinal manifestations like nausea and vomiting, as well as hepatotoxicity [8, 9]. Therefore, developing safer and more effective therapeutic strategies against MASH and its complications remains an urgent priority.

The pathogenesis and progression of MASH are significantly driven by gut microbiota dysbiosis and intestinal barrier dysfunction [10, 11]. The liver and gut interact bidirectionally through the gut‐liver axis– a regulatory network initiating at the intestinal barrier, mediated via the portal circulation and biliary system [12]. Notably, disruption of the intestinal barrier serves as a pivotal pathogenic trigger in MASH development [13]. Thereby, it allows pro‐inflammatory inducer lipopolysaccharide (LPS) to translocate to the liver via the portal vein, subsequently promoting the production of inflammatory cytokines and forming a vicious cycle of “gut leakage‐liver injury” [14]. Furthermore, these mediators may further cross the blood‐brain barrier (BBB) and penetrate key cerebral areas including the hippocampus to exacerbate neuroinflammation [15]. Therefore, restoring the integrity of the intestinal barrier has emerged as a key upstream therapeutic strategy for intervening in MASH pathogenesis. Interleukin‐22 (IL‐22), a cytokine secreted by innate lymphoid cells and Th17/22 cells, exerts barrier‐protective functions through binding to its specific receptors [16]. Previous studies have indicated that high‐fat diet (HFD) suppressed intestinal IL‐22 expression, whereas exogenous recombinant IL‐22 administration activated the STAT3 signaling pathway via engagement with intestinal epithelial receptors (IL‐22Ra1), thereby effectively ameliorating hepatic steatosis, inflammation, and fibrosis in MASH [17, 18]. However, the therapeutic potential of IL‐22 has been severely undermined by its safety concerns. Chronic systemic administration of IL‐22 (e.g., IL‐22Fc) induced excessive proliferation of the skin and intestines, eczema or gastrointestinal dysfunction [19, 20, 21]. This presents a clear clinical challenge: how to harness the powerful therapeutic benefits of IL‐22 in the gut while avoiding its severe off‐target toxicities.

To overcome this challenge, an ideal delivery system needs to precisely confine the production of IL‐22 to the intestinal tract, necessitating on‐demand administration. Recent advances in engineered probiotics offer a promising approach, with bacteria such as *Escherichia coli* Nissle 1917 (EcN1917) serving as live therapeutics [22, 23, 24, 25]. To enhance the regulatory precision of engineered bacteria, optogenetic systems have been implemented, enabling real‐time, spatiotemporally precise manipulation of probiotics [26]. However, tissue photon scattering and absorption phenomena severely restrict light penetration depth, substantially limiting their applicability in deep‐seated tissues such as the gut [27]. In contrast, sonogenetics utilizes non‐invasive, high‐penetration ultrasound to regulate gene expression, emerging as a compelling alternative to optogenetics [28, 29, 30].

Here, we for the first time innovatively combine these concepts to develop an oral, sonogenetically engineered probiotic system. We engineered EcN1917 to produce IL‐22 under the control of an ultrasound‐responsive promoter, and encapsulated the engineered bacteria in an inulin hydrogel. This dual‐guarantee mechanism of “smart encapsulation plus ultrasound‐controlled release” ensures on‐demand delivery of the factor at specific times and locations. Metabolomic sequencing and Western blot (WB) analysis further revealed that the development of MASH was accompanied by overactivation of the PI3K‐AKT‐mTOR signaling pathway. Our therapeutic strategy, by inhibiting the overactivation of this pathway at its source, demonstrated multi‐dimensional synergistic effects: remodeling the gut microbiota, repairing the intestinal barrier, improving hepatic pathology and protecting cognitive function. Notably, this study provided the first experimental evidence that modulating the gut‐liver axis reduced LPS levels in the portal vein and subsequently decreased hepatic IL‐6 production, enabling systemic benefits from metabolic disorders to neurological function. This work not only provides a novel technological platform for MASH treatment but also further establishes a trinity precision medicine paradigm of “physical regulation—biological therapy—systemic intervention”. We anticipate that this strategy will vigorously promote the application of sonogenetic technology in metabolic diseases, paving a new path for innovating future disease treatment modalities.

## 2 Results

### 2.1 Design and Characterization of a US‐Activated Sonogenetic Probiotic System

To develop a sonogenetic bacterial therapy for the treatment of MASH, we selected EcN1917 as the engineering chassis due to its favorable therapeutic safety and genetic modification compatibility [23]. Firstly, we successfully constructed an engineered strain (Lux‐EcN) expressing LuxCDABE luciferase (Figure 1a), thereby providing a real‐time monitoring capability for sonogenetic regulation. Under the control of the plasmid's thermosensitive regulatory circuit (Figure S1), elevating the temperature to 42°C initiated LuxCDABE reporter gene expression via activation of the Hsh promoter.

**FIGURE 1:**
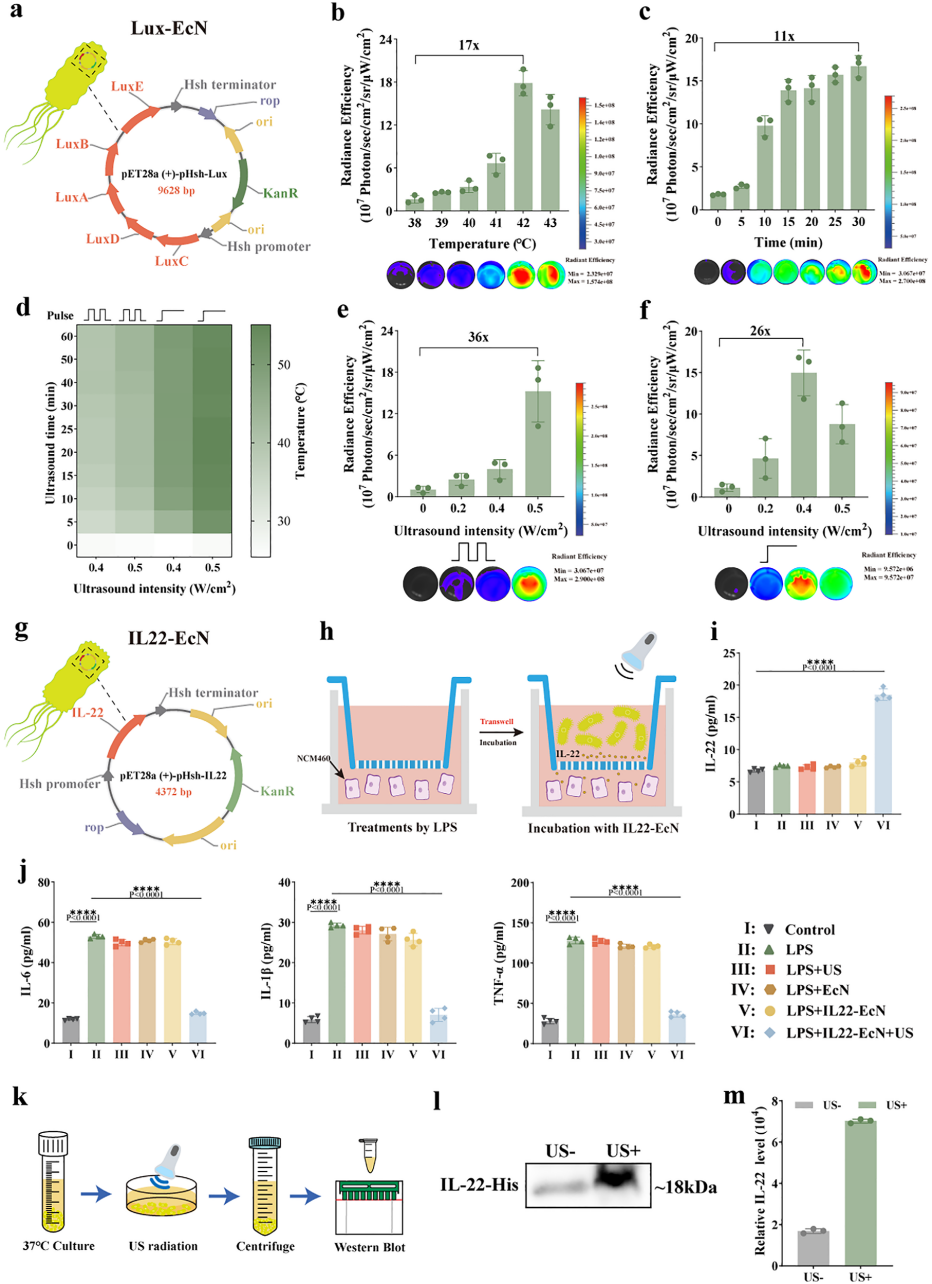
Optimization and anti‐inflammatory efficacy of an US‐Triggered sonogenetic probiotic system. (a) Schematic diagram of the constructed pET28a (+)‐pHsh‐Lux (9628 bp) plasmid in engineered Lux‐EcN. (b) Quantitative analysis of transgene expression in engineered Lux‐EcN at different temperatures (n = 3 independent experiments). (c) Transgene expression level under different durations of thermal induction (n = 3 independent experiments). (d) Heatmap of bacterial suspension temperature and ultrasonic parameters. (e and f) Transgene expression under different ultrasound intensities using 1:1 pulsed‐mode (e) and continuous ultrasound (f) (n = 3 independent experiments). (g) Schematic diagram of the constructed pET28a (+)‐pHsh‐IL22 (4372 bp) plasmid in engineered IL22‐EcN. (h) Schematic diagram of the Transwell co‐culture system used to study ultrasound‐regulated IL22 expression by IL22‐EcN in vitro. (i) IL‐22 expression under different treatments in a Transwell co‐culture system measured by ELISA (n = 4 biologically independent samples). (j) Levels of IL‐6, IL‐1β and TNF‐α under different treatment groups quantified by ELISA (n = 4 biologically independent samples). (k) Schematic of WB detection of ultrasound‐induced IL‐22 expression in IL22‐EcN. (l and m) WB (l) and quantitative analysis (m) of IL‐22 expression in IL22‐EcN after ultrasound stimulation (n = 3 independent experiments). Data in b, c, e, f, i, j and m are presented as means ± SD. P values for i and j were calculated by one‐way ANOVA followed by Tukey's multiple comparison test. ****p < 0.0001.

To achieve non‐invasive ultrasound regulation of the sonogenetic system, we cultured Lux‐EcN under different temperatures and observed the peak bioluminescence signal intensity was at 42°C (a generally safe temperature), increasing by approximately 17‐fold, demonstrating efficient activation of target gene transcription without thermal damage (Figure 1b). In addition, gene expression levels increased with prolonged thermal induction time (Figure 1c). Through screening of the acoustic parameters (ultrasound intensity and duration), we identified that an intensity of 0.5 W/cm^2^ with a 1:1 pulse mode (1s on /1s off) for 30 min could stably maintain the temperature at 42°C (Figure 1d), thereby effectively activating this sonogenetic system. This pulsed mode achieved 36‐fold higher gene expression than non‐sonicated group and outperformed continuous ultrasound at 0.4 W/cm^2^ or 0.5 W/cm^2^ (Figure 1e,f).

Subsequently, we transformed the pET28a (+)‐pHsh‐IL22 plasmid into EcN1917, successfully obtaining the engineered strain expressing IL‐22 (IL22‐EcN) (Figure 1g). To validate in vitro whether ultrasound‐regulated IL22‐EcN could express IL‐22 and exert anti‐inflammatory effects, we employed the Transwell co‐culture system. IL22‐EcN were placed in the upper chamber while LPS‐induced inflammatory normal‐derived colon mucosa (NCM460) cells were placed in the lower chamber (Figure 1h). After ultrasonic stimulation (0.5 W/cm^2^, 1s on/1s off, 30 min) and co‐culture for 12 h, ELISA assays revealed that IL‐22 secretion significantly suppressed pro‐inflammatory cytokines (IL‐6, IL‐1β and TNF‐α) (Figure 1i,j). WB further confirmed that after treating the IL22‐EcN bacterial suspension with ultrasound (0.5 W/cm^2^, 1s on/1s off, 30 min), substantial IL‐22 protein secretion was detected in the culture supernatant. In contrast, the non‐sonicated control group showed only minimal IL‐22 expression due to the basal “leakage effect” of the Hsh promoter (Figure 1k–m). These results indicated that ultrasound could effectively activate the expression of IL‐22 in IL22‐EcN, suggesting its promising potential for treating inflammatory diseases.

Safety assessment showed that IL22‐EcN bacterial suspension was subjected to ultrasound treatment at 0.5 W/cm^2^ (1s on/1s off, 30 min). IL22‐EcN survival assessed by CCK‐8 assay revealed no significant difference in survival rate between the sonicated group and the non‐sonicated control group (Figure S2a). Growth curve analysis further demonstrated that ultrasound treatment did not inhibit bacterial growth (Figure S2b). Additionally, live/dead bacterial viability staining showed that IL22‐EcN maintained high viability after ultrasound stimulation, with no obvious bacterial lysis or cell death observed under fluorescence microscopy (Figure S2c). We also assessed the viability of various cell types after ultrasound treatment using the CCK‐8 assay. The cells tested included intestinal epithelial cells (IECs), colorectal cancer cells (HCT‐8), hepatocytes (AML‐12), renal tubular epithelial cells (HK‐2) and human skin fibroblasts (HSF). The results indicated that this ultrasound parameter caused no significant adverse effects on the viability of either normal tissue cells or tumor cells (Figure S2d,e), demonstrating good biosafety of the ultrasound treatment.

### 2.2 Preparation and Characterization of IL22‐EcN/Gel

To further enhance the bioavailability of the engineered probiotic in the gastrointestinal tract, we combined IL22‐EcN with inulin, a food additive approved by FDA, to prepare a novel oral engineered probiotic hydrogel formulation. IL22‐EcN was cultured at 37°C in kanamycin‐supplemented LB liquid medium. Scanning electron microscopy (SEM) images revealed that IL22‐EcN exhibited a typical rod‐shaped morphology, with a length of approximately 1.2‐2.0 µm and a diameter of approximately 0.5‐0.6 µm (Figure 2a). To prepare the probiotic hydrogel, IL22‐EcN was mixed into an inulin solution that had been heated and naturally cooled to 37°C, and then left standing at room temperature for 12 h to form the IL22‐EcN/Gel (Figure 2b). The microstructure of the inulin hydrogel was characterized using SEM. As shown in Figure 2c, the hydrogel exhibits a loose, interconnected network structure with non‐uniform pore size distribution. SEM images of IL22‐EcN/Gel at different magnifications confirmed IL22‐EcN was effectively encapsulated in the hydrogel (Figure 2c). Rheological tests demonstrated that IL22‐EcN/Gel exhibited distinct “shear‐thinning” behavior with increasing shear rate (0.1 s^−^^1^ to 100 s^−^^1^), confirming its characteristic medium‐viscosity properties (Figure 2d). The significantly greater storage modulus (G′) than the loss modulus (G″) showed that IL22‐EcN/Gel was a predominantly elastic material rather than a flowing liquid, and a stable three‐dimensional cross‐linked network has been formed (Figure 2e).

**FIGURE 2:**
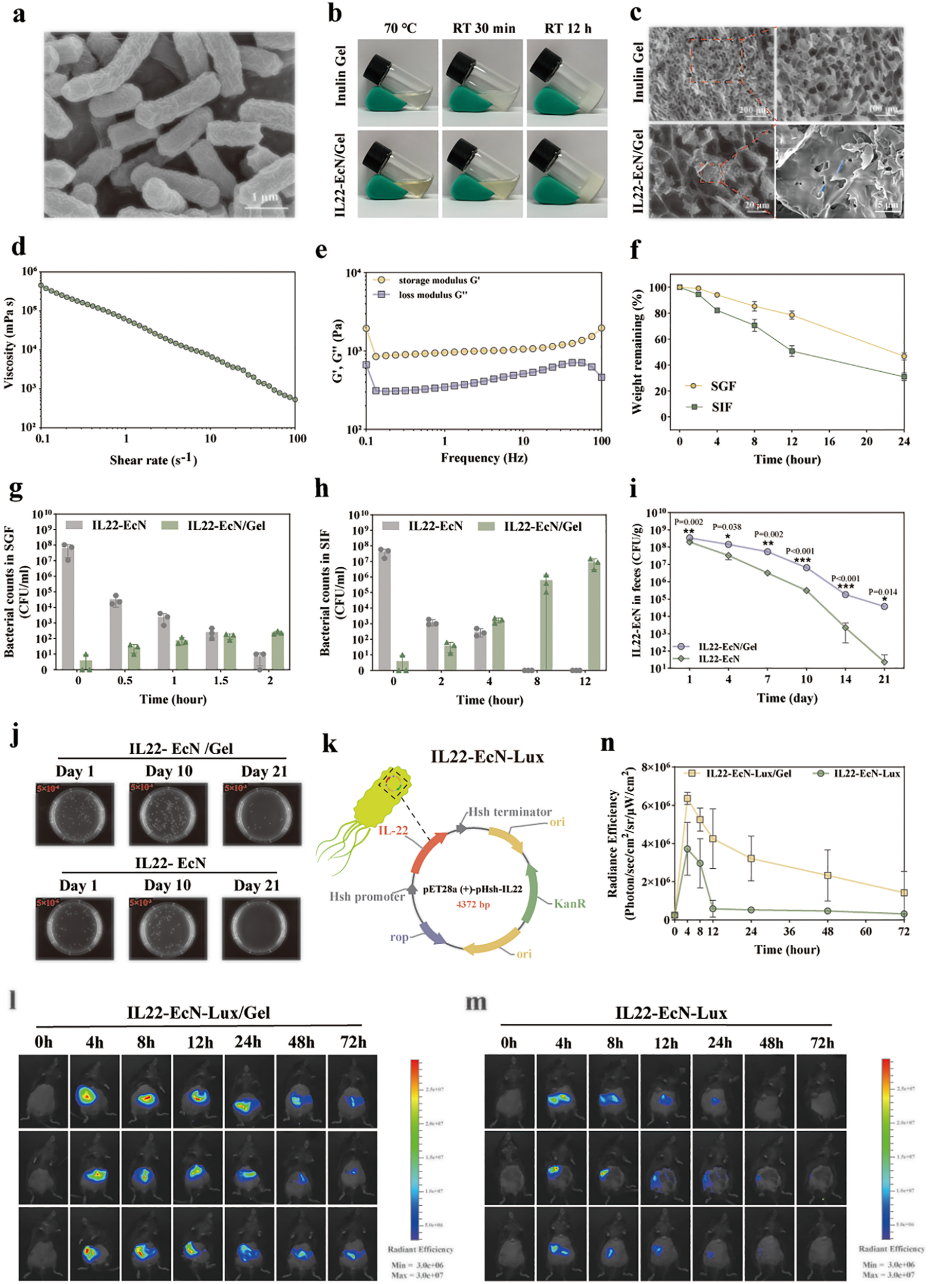
Characterization and gastrointestinal colonization of IL22‐EcN/Gel. (a) Representative SEM images of IL22‐EcN. Scale bar: 1 µm. (b) Digital photographs of the gelation states of inulin hydrogel and IL22‐EcN/Gel under different conditions. (c) SEM images of inulin hydrogel and IL22‐EcN/Gel at different magnifications. Scale bar: 200 µm and 20 µm; inset of 100 µm and 5 µm. (d) Shear‐thinning behavior of IL22‐EcN/Gel (shear rate from 0.1 s^−^^1^ to 100 s^−^^1^). (e) Storage modulus (G′) and loss modulus (G″) of IL22‐EcN/Gel (frequency from 0.1 Hz to 100 Hz). (f) In vitro degradation of IL22‐EcN/Gel in SGF and SIF. (g) Quantitative analysis of CFU for the release of IL22‐EcN in SGF in vitro. (h) Quantitative analysis of CFU for the release of IL22‐EcN in SIF in vitro. (i) Quantitative analysis of viable IL22‐EcN in mice feces at different time points (n = 3 mice per group at each time point). (j) Representative photographs of agar plate demonstrating viable IL22‐EcN in mice feces at different time points. (k) Schematic diagram of the constructed pET28a (+)‐pHsh‐IL22 (4372 bp) plasmid in engineered IL22‐EcN‐Lux. (l) Bioluminescence images of the gastrointestinal tract in MASH mice at different time points after oral administration of IL22‐EcN‐Lux/Gel. (m) Bioluminescence images of the gastrointestinal tract in MASH mice at different time points after oral administration of IL22‐EcN‐Lux. (n) Quantitative evaluation of bioluminescence signal intensity of the IL22‐EcN‐Lux/Gel and the IL22‐EcN‐Lux mice. Data in f, g, h, i and n are presented as means ± SD from n = 3 biological replicates. P values were calculated by two‐tailed unpaired student's t‐test. *p < 0.05, **p < 0.01 and ***p < 0.001.

We also evaluated the degradation of IL22‐EcN/Gel in simulated gastric fluid (SGF) and simulated intestinal fluid (SIF) (Figure 2f). The hydrogel exhibited significantly higher degradation in SIF (about 70% degradation within 24 h) compared to SGF (about 54% degradation within 24 h), likely due to synergistic enzymatic activity and alkaline conditions that might promote hydrogel swelling or enzymatic degradation. Next, we assessed the release of IL22‐EcN and IL22‐EcN/Gel in SGF and SIF. In SGF, the viable bacteria count in the IL22‐EcN/Gel group gradually increased with the degradation of inulin hydrogel within the first 2 h (Figure 2g), indicating that the hydrogel encapsulation effectively alleviated the killing effect of gastric acid on bacteria and enabled sustained bacterial release. Similarly, in SIF, the viable bacteria count in the IL22‐EcN/Gel group also steadily increased within the first 12 h due to the continuous degradation of the hydrogel (Figure 2h). In contrast, the unencapsulated IL22‐EcN group showed a rapid viability loss in both SGF and SIF, further highlighting the crucial role of hydrogel in bacterial protection.

To evaluate intestinal colonization efficiency, healthy mice received 1×10^8^ CFUs of IL22‐EcN or IL22‐EcN/Gel via oral gavage, followed by quantitative fecal colony counting of viable bacteria. Notably, the IL22‐EcN/Gel group demonstrated superior fecal bacterial loads and prolonged gastrointestinal persistence for more than 14 days compared to the unencapsulated IL22‐EcN group (Figure 2i,j), indicating that inulin hydrogel encapsulation significantly improved survival rate and retention capacity of engineered probiotics in the gut.

### 2.3 Gastrointestinal Colonization of IL22‐EcN‐Lux/Gel In Vivo

The complex gastrointestinal environment (such as pH, digestive enzymes, bile salts) poses multiple challenges to oral delivery systems, not only causing significant inactivation of probiotics during delivery but also severely limiting their intestinal colonization efficiency [31]. Therefore, to systematically evaluate the gastrointestinal retention properties of the inulin hydrogel in vivo, as shown in Figure 2k, we constructed an IL‐22‐expressing strain integrated with the LuxCDABE reporter gene (IL22‐EcN‐Lux). IL22‐EcN‐Lux and IL22‐EcN‐Lux/Gel were respectively administered orally to MASH mice. In vivo imaging revealed that the IL22‐EcN‐Lux/Gel group exhibited ∼7‐fold stronger bioluminescence signal intensity compared to the IL22‐EcN‐Lux group at multiple time points post‐administration (Figure 2l–n). It is worth noting that the bioluminescence duration of the IL22‐EcN‐Lux/Gel group was significantly prolonged, and it could still be detected 72 h after administration, indicating that inulin hydrogel effectively functioned as a drug delivery to prolong intestinal retention of engineered probiotics. To further investigate the biodistribution of IL22‐EcN‐Lux/Gel, major organs (heart, liver, spleen, lung, kidney, brain and gastrointestinal tract) were harvested 72 h after intragastric administration for in vitro fluorescence imaging. In agreement with in vivo findings, the IL22‐EcN‐Lux/Gel group exhibited significantly stronger and more persistent bioluminescence signal in the gastrointestinal tract compared to the IL22‐EcN‐Lux group, with signals progressively migrating toward the cecum and colon (Figure S3a,b). Critically, no bioluminescence was detected in extraintestinal organs (heart, liver, spleen, lung, kidney and brain), confirming absence of systemic accumulation (Figure S3c). Therefore, the encapsulation of inulin hydrogel further enhanced site‐specific adhesion and retention of engineered probiotics in the intestinal tract.

Subsequently, in order to monitor gene expression of this sonogenetic system in vivo, we orally administered Lux‐EcN/Gel (synthesized as described above) to C57BL/6 mice that had been fed a choline‐deficient high‐fat diet (CDHFD) for 16 weeks. The abdominal region was exposed to ultrasound (0.5 W/cm^2^, 1s on/1s off) with varying exposure durations. During ultrasound irradiation, infrared thermography was used to monitor abdominal surface temperature in real‐time to ensure stable maintenance at 42°C (Figure S4). In vivo bioluminescence imaging revealed gene expression levels increased in a time‐dependent manner by ultrasound irradiation (Figure S5a–c).

### 2.4 Biosafety Evaluation of IL22‐EcN/Gel

In this study, healthy 7‐week‐old mice were treated with IL22‐EcN/Gel via oral gavage, followed by abdominal ultrasound treatment (0.5 W/cm^2^, 1s on/1s off) for 30 min to evaluate its biosafety (Figure S6a). On the third, seventh and 30th days after intragastric administration, hematoxylin and eosin (H&E) staining results showed no damage to major organs (heart, liver, spleen, lung, kidney, intestine and brain) in IL22‐EcN/Gel combined with ultrasound treatment (Figure S6b,c). Complete blood count (CBC) and serum biochemical indicators, including white blood cells (WBC), red blood cells (RBC), platelets (PLT), hemoglobin (HGB), alanine aminotransferase (ALT), aspartate aminotransferase (AST), albumin (ALB), blood urea nitrogen (BUN), uric acid (UA), and creatinine (CREA), remained within normal ranges and showed no significant differences from the control group (Figure S6d–h). These results demonstrated that IL22‐EcN/Gel exhibited favorable biosafety in healthy mice under ultrasound activation.

### 2.5 Therapeutic Effects of IL22‐EcN/Gel + US Treatment in MASH

Since choline is a key substrate for lipoprotein secretion by the liver, its deficiency leads to intrahepatic lipid accumulation, thereby exacerbating MASH [32]. We establish a MASH model by feeding C57BL/6 male mice (7 weeks old, ∼22 g) CDHFD for 16 weeks, and then divided them into seven different groups: I) normal chow diet (NCD), II) CDHFD, III) CDHFD + US irradiation, IV) CDHFD + EcN, V) CDHFD + IL22‐EcN, VI) CDHFD + IL22‐EcN/Gel, VII) CDHFD + IL22‐EcN/Gel + US irradiation (Figure 3a).

**FIGURE 3:**
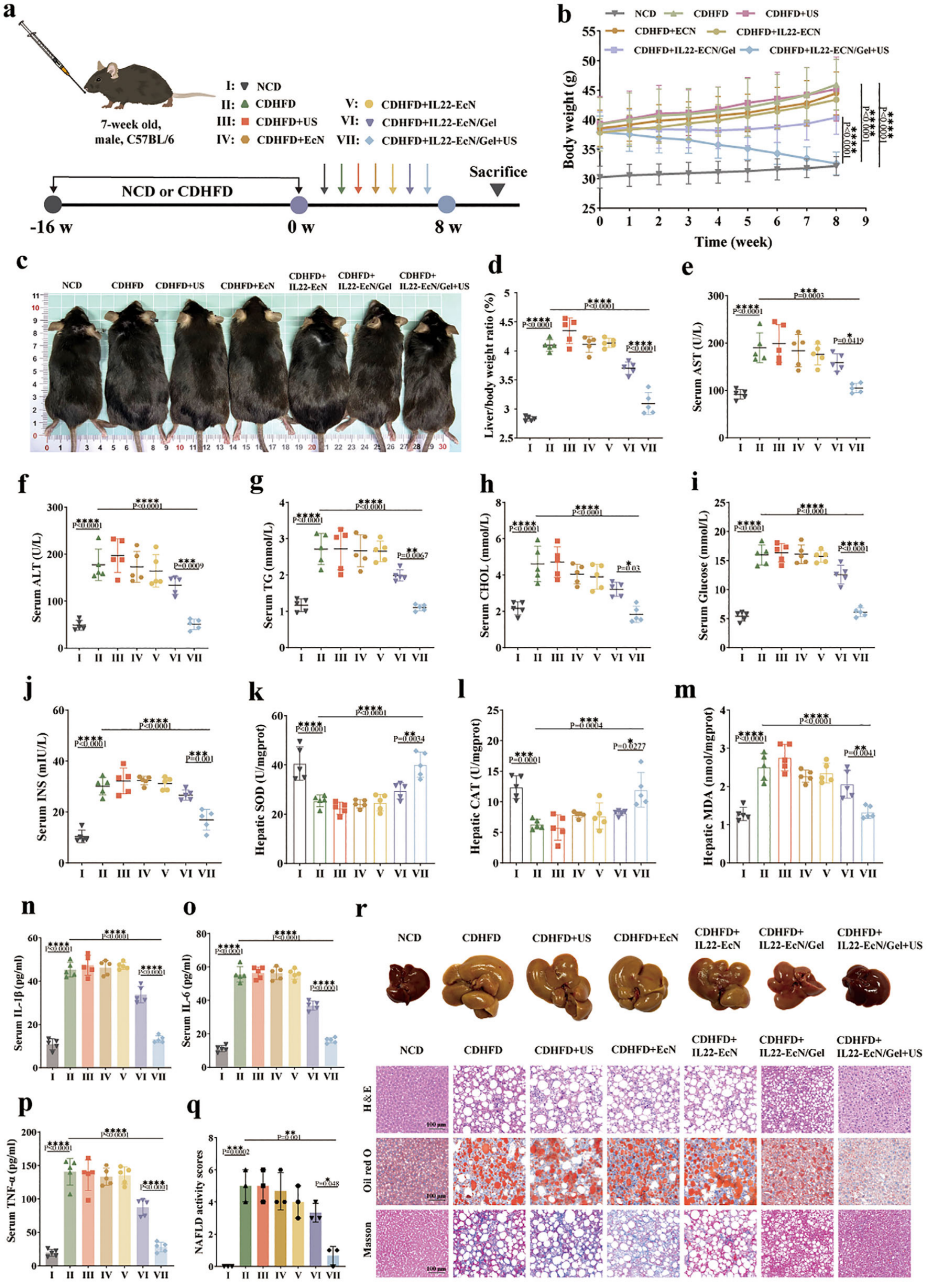
The therapeutic effect of IL22‐EcN/Gel combined with US irradiation against CDHFD‐induced MASH. (a) Schematic diagram of treatment regimens for different groups. (b) Body weight change curves of mice in different groups during the treatment period. (c) Digital photographs of the gross morphology of mice from different treatment groups. (d) Liver/body weight ratio of mice in different groups. (e) Serum AST. (f) Serum ALT. (g) Serum TG. (h) Serum CHOL. (i) Serum Glucose. (j) Serum INS. (k‐m) Hepatic SOD (k), hepatic CAT (l) and hepatic MDA (m). (n‐p) Serum IL‐1β (n), IL‐6 (o) and TNF‐α (p). (q) NAFLD activity scores (NAS) in different groups (n = 3 per group). (r) Typical liver morphology images and corresponding H&E, Oil Red O and Masson staining sections. Scale bar: 100 µm. Data in b, d, e, f, g, h, i, j, k, l, m, n, o and p are presented as means ± SD from n = 5 biological independent samples. P values in b were calculated by two‐way ANOVA. P values in d‐q were calculated by one‐way ANOVA followed by Tukey's multiple comparison test. *p < 0.05, **p < 0.01, ***p < 0.001 and ****p < 0.0001.

Compared with the CDHFD group, group VII (CDHFD + IL22‐EcN/Gel + US irradiation) showed a decrease in body weight during the treatment period and ultimately approached the body weight of the NCD group, while the body weight in the other groups increased to varying degrees (Figure 3b). Digital photographs capturing the gross morphology of mice after treatment further supported these findings (Figure 3c). Food intake recorded during the treatment period was significantly lower in group VII than in the other six groups (Figure S7a), suggesting a potential role of IL‐22 in regulating feeding behavior [17]. After euthanasia, the liver and spleen were excised and weighed. The results demonstrated that group VII had significantly lower liver weight, spleen weight and liver/body weight ratio compared to the other five groups (groups II‐VI), approaching the NCD group (Figure S7b,c; Figure 3d). We observed IL22‐EcN/Gel combined with US irradiation treatment significantly alleviated CDHFD‐induced liver injury, hepatic lipid accumulation and hyperglycemia, manifested by significantly reduction in serum aspartate aminotransferase (AST), alanine aminotransferase (ALT), serum triglycerides (TG), serum total cholesterol (CHOL), hepatic TG, hepatic CHOL, blood glucose and serum insulin (Figure 3e–j; Figure S7d,e). Furthermore, this combined treatment demonstrated greater efficacy than the IL22‐EcN/Gel group. Consistent with these improvements, hepatic superoxide dismutase (SOD) and catalase (CAT) levels were restored after treatment, while malondialdehyde (MDA) levels were significantly decreased (Figure 3k–m), indicating a substantial amelioration of oxidative stress.

To assess inflammation levels, we quantified serum concentrations of classical pro‐inflammatory cytokines (IL‐1β, IL‐6 and TNF‐α) using ELISA. As shown in Figure 3n–p, the CDHFD group exhibited significantly elevated levels of IL‐1β, IL‐6 and TNF‐α compared to the NCD group. As anticipated, these pro‐inflammatory cytokines were markedly reduced after combined treatment. Histopathological analysis of liver tissues was consistent with these findings: H&E, Oil Red O and Masson staining further demonstrated that IL22‐EcN/Gel + US treatment substantially ameliorated hepatic steatosis, inflammatory infiltration and fibrosis in MASH mice (Figure 3q,r). In contrast, IL22‐EcN/Gel treatment (Group VI, without US irradiation) showed limited therapeutic effects and failed to fully reverse hepatic lipid deposition, inflammation, fibrosis and oxidative stress damage. These key observations were also validated in the high‐fat, high‐cholesterol (HFHC) model (Figure S8). Overall, these results highlighted the critical role of the spatiotemporally controllable sonogenetic system in achieving effective MASH intervention.

### 2.6 Regulatory Effects of IL22‐EcN/Gel + US Treatment on Gut Microbiome and Intestinal Barrier

The liver and gut communicate bidirectionally through the “gut‐liver axis”, which originates at the intestinal barrier and terminates at the portal vein, biliary tract and liver [12]. Both gut microbiota dysbiosis and impaired intestinal barrier contribute significantly to the development and progression of MASH [13]. To explore whether our sonogenetic system modulates the gut microbiota, we conducted 16S ribosomal RNA (rRNA) gene sequencing on samples from the NCD group, CDHFD group and the group treated with IL22‐EcN/Gel combined with US. Principal coordinates analysis (PCoA) and non‐metric multidimensional scaling (NMDS) revealed distinct microbial compositions among the three groups (Figure 4a,b). At the phylum level, CDHFD induction led to an increase in the relative abundance of Firmicutes and a decrease in Bacteroidota, which collectively elevated the Firmicutes/Bacteroidota (F/B) ratio (Figure S9a–d). Although not statistically significant, IL22‐EcN/Gel combined with US treatment tended to reverse this trend, reducing Firmicutes and increasing Bacteroidota (P = 0.0754; P = 0.0842). Further analysis at the genus and family levels showed significant changes in the gut microbiota composition among the three groups. At the genus level, the treated group exhibited a significant increase in Alloprevotella (Bacteroidota phylum), a known producer of short‐chain fatty acids (SCFAs) that helps maintain gut microecological balance [33], alongside a significant decrease in Faecalibaculum (Firmicutes phylum), a genus linked to driving hepatic inflammation [34] (Figure 4c,d; Figure S10a). Similarly, at the family level, we observed a significant reduction in Lachnospiraceae (previously associated with MASH progression [12]) and a marked increase in Prevotellaceae in the treated group, which is known to contribute to SCFAs production [35] (Figure 4e,f; Figure S10b). Linear discriminant analysis effect size (LEfSe) further verified the above findings (Figure 4g,h). Therefore, the combination of IL22‐EcN/Gel and US treatment led to shifts in the gut microbiota composition, marked by a significant decrease in pathogenic bacteria and a concurrent enrichment of beneficial bacteria.

**FIGURE 4:**
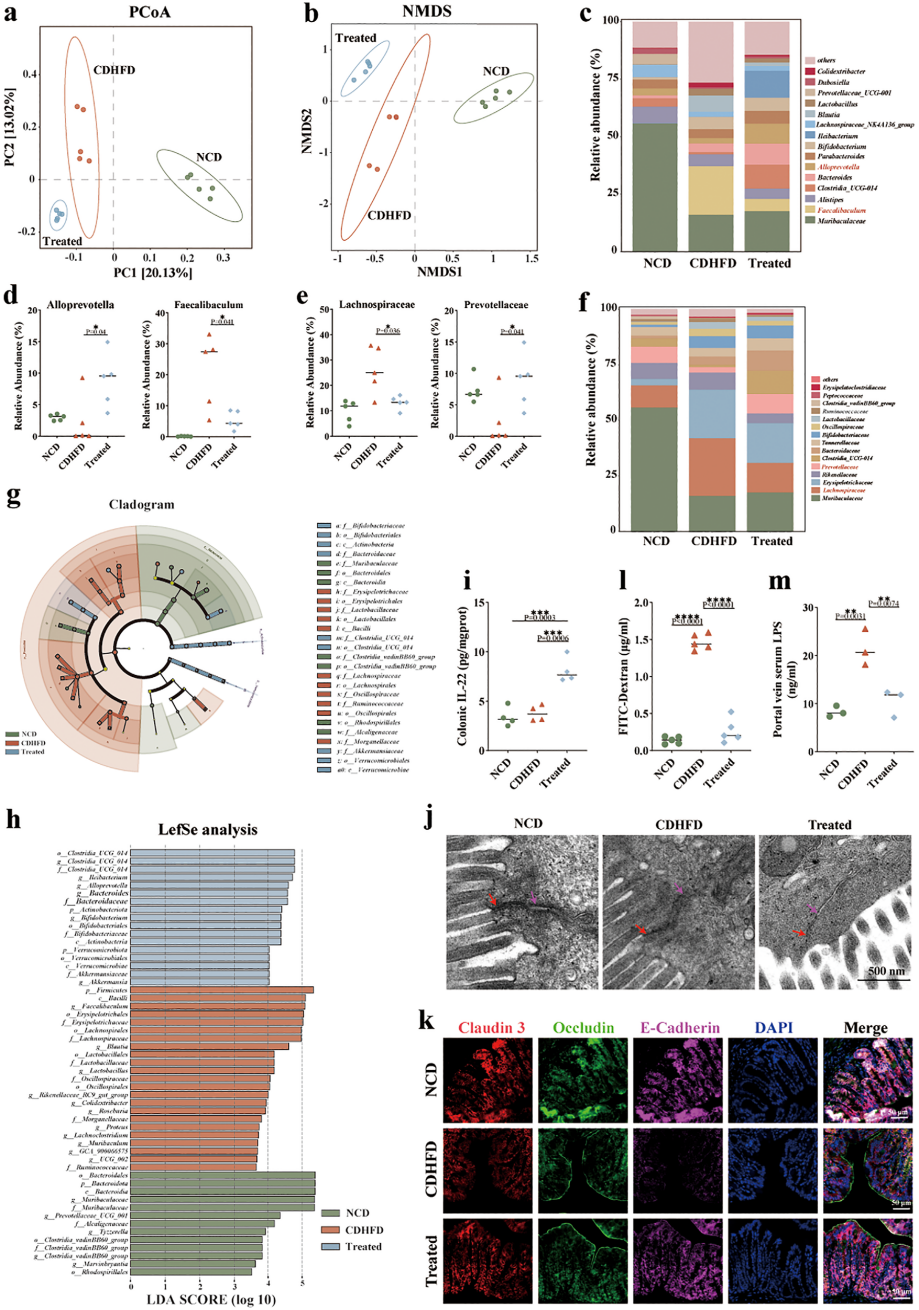
Regulatory effects of IL22‐EcN/Gel + US treatment on intestinal barrier and gut microbiome. (a, b) Fecal microbial compositions visualized by PCoA (a) and NMDS (b). (c) Relative abundance of gut microbiota at the genus level from different groups. (d) Alloprevotella and Faecalibaculum relative abundance at the genus level. (e) Lachnospiraceae and Prevotellaceae relative abundance at the family level. (f) Relative abundance of gut microbiota at the family level from different groups. (g) Annotated cladogram of discriminative bacteria from different groups by LEfSe analysis. (h) Linear discriminant analysis (LDA) scores of discriminative bacteria from different groups by LEfSe analysis. (i) IL‐22 levels in the colon from different groups of mice measured by ELISA (n = 4 biologically independent samples). (j) Representative TEM images of mice colonic epithelium. Scale bar: 500 nm. Red and pink arrows indicate tight junctions and adherens junctions, respectively. (k) Representative colon images of immunofluorescence staining for Claudin 3 (red), Occludin (green), E‐Cadherin (pink) and DAPI (blue). (l) FITC‐dextran permeability assay from different groups of mice (n = 5 biologically independent samples). (m) LPS levels in portal vein serum from different groups of mice (n = 3 biologically independent samples). a‐h were n = 5 biological independent samples. Statistical analyses were calculated by one‐way ANOVA followed by Tukey's multiple comparison test (i‐m) and two‐tailed unpaired Student's t‐test (d and e). *p < 0.05, **p < 0.01, ***p < 0.001 and ****p < 0.0001.

Then, to determine whether gut microbiota remodeling by IL22‐EcN/Gel combined with US treatment subsequently altered the metabolic profile, we performed untargeted metabolomics analysis on fecal samples from the NCD group, CDHFD group and treatment group (IL22‐EcN/Gel + US). Partial least squares discriminant analysis (PLS‐DA) displayed distinct clustering in metabolomic profiles, indicating significant inter‐group differences and strong intra‐group homogeneity (Figure S11a). A clustered heatmap, generated from the intersection of differential metabolites (defined as the treatment‐reversal metabolites) between the CDHFD vs. NCD and CDHFD vs. treatment, displayed distinct differential metabolic profiles across the groups (Figure S11b). KEGG pathway enrichment analysis further identified “Choline metabolism in cancer” as one of the most significantly enriched pathways among the treatment‐reversal metabolites (Figure S11c). Notably, choline metabolism is recognized as a core pathway critically involved in the pathogenesis and progression of MASLD/MASH [36]. Using screening criteria of *p* < 0.05 and fold change > 2, we detected 3646 differentially abundant metabolites between the NCD and CDHFD groups, among which 1373 were upregulated and 2273 were downregulated. Notably, IL22‐EcN/Gel combined with US treatment significantly changed the metabolic profile, with 747 upregulated and 858 downregulated metabolites detected (Figure S11d–f). KEGG pathway enrichment analysis revealed that compared to the NCD group, the CDHFD group exhibited enrichment in pathways involved in linoleic acid metabolism, neuroactive ligand‐receptor interaction and choline metabolism in cancer (Figure S11g). These metabolic pathways, which regulated processes such as lipid metabolism, neuroimmunity, inflammation and oxidative stress, have been confirmed to be implicated in the pathogenesis of MASH [6, 37, 38, 39]. Interestingly, when comparing the CDHFD group to the treatment group, the enrichment in these aforementioned metabolic pathways was reduced or eliminated (Figure S11h). Consequently, our findings indicated through gut microbiota remodeling, IL22‐EcN/Gel combined with US treatment thereby normalized MASH‐related metabolic pathways.

Meanwhile, considering that gut microbiota dysbiosis is a key driver of intestinal barrier impairment [32, 40], we investigated the regulatory effects of IL22‐EcN/Gel combined with US treatment on the damaged intestinal barrier. As shown in Figure 4i, colon IL‐22 levels in the treatment group (IL22‐EcN/Gel + US) were significantly higher than in both the NCD and CDHFD group, confirming that this sonogenetic system enabled targeted delivery and efficient release of IL‐22 in the colon. Similarly, transmission electron microscopy (TEM) revealed significant disruption of tight and adherens junctions between colonic epithelial cells in the CDHFD group, as indicated by the arrows in Figure 4j. In contrast, treatment with IL22‐EcN/Gel combined with US restored their structural integrity. This was further confirmed by immunofluorescence staining of the colon (Figure 4k), and the fluorescence signals of tight junction proteins Claudin 3 (red), Occludin (green) and adherens junction protein E‐Cadherin (pink) in the colonic tissues of the CDHFD group were attenuated. Research has shown that these proteins play crucial roles in maintaining intestinal barrier integrity [41]. Conversely, following treatment with IL22‐EcN/Gel combined with US irradiation, the fluorescence signals were restored, indicating restoration of the intestinal barrier. The fluorescein isothiocyanate (FITC)‐dextran permeability assay also showed a significant reduction in intestinal permeability in CDHFD group after treatment (Figure 4l). These results suggested that IL22‐EcN/Gel combined with US treatment improved intestinal barrier function. Collectively, these results strongly indicated that IL22‐EcN/Gel combined with US treatment significantly reversed these abnormal metabolic characteristics, thereby effectively restoring intestinal barrier integrity and improving the gut microbiota.

### 2.7 Validation of the Anti‐Inflammatory Effect in an In Vitro Gut‐liver Axis Co‐Culture Model

To further validate the above observations, we employed a Transwell co‐culture system to simulate the gut‐liver axis interaction. Mouse primary colonic epithelial cells (CECs) and IL22‐EcN were placed in the upper chamber, while LPS‐induced mouse primary hepatocytes (MPH) were placed in the lower chamber (Figure S12a). The co‐culture system was subjected to ultrasound stimulation (0.5 W/cm^2^, 1s on/1s off, 30 min) and then incubated for 12 h. ELISA results showed that, compared with the other groups, the IL‐22 production in the upper chamber was significantly increased in the IL22‐EcN+US treatment group, while the levels of pro‐inflammatory cytokines (IL‐6, IL‐1β, and TNF‐α) in the culture supernatant of hepatocytes in the lower chamber were markedly decreased (Figure S12b–e). These findings indicated that IL22‐EcN + US treatment could effectively suppress hepatocyte inflammatory responses via the gut‐liver axis.

### 2.8 Therapeutic Mechanism of IL22‐EcN/Gel + US Treatment in MASH

We then measured LPS levels in the portal vein serum, a key gut‐derived factor that propagates inflammation along the gut‐liver axis. Given this observation that the CDHFD group showed significantly elevated LPS levels, whereas the treatment group restored to near‐normal (Figure 4m), prompting us to investigate the underlying mechanisms responsible for the hepatic alterations. Correspondingly, we performed untargeted metabolomics profiling on serum samples from the NCD group, CDHFD group and treatment group (IL22‐EcN/Gel + US). Principal component analysis (PCA) revealed distinct clustering in metabolomic profiles among the three groups, with high intra‐group metabolic homogeneity (Figure 5a). However, when applying the same intersection approach to serum metabolomics, we obtained only a small number of metabolites (Figure S13a,b). Given that serum metabolomics is more representative of systemic metabolic reprogramming, and to prevent the loss of potentially important metabolites under stringent filtering criteria, we consequently switched to a union strategy for treatment‐reversal metabolites, thereby ensuring a more comprehensive profiling. Clustering heatmap and KEGG enrichment analysis further revealed that the treatment‐reversed metabolites were predominantly enriched in the PI3K‐AKT‐mTOR signaling pathway (Figure 5b,c). Metabolomic analysis identified 693 differentially abundant metabolites between the NCD and CDHFD groups (screening criteria: *p* < 0.05, fold change > 2), comprising 297 upregulated and 396 downregulated metabolites. In contrast, 321 differentially abundant metabolites were detected between the CDHFD and treatment groups, with 180 upregulated and 141 downregulated metabolites (Figure 5d–f). KEGG pathway analysis comparing the NCD and CDHFD groups revealed significant enrichment in pathways such as histidine metabolism and steroid hormone biosynthesis (Figure 5g). It was reported that these metabolic pathways were involved in the progression of MASH by influencing lipid metabolism, hepatic inflammation and insulin resistance [42, 43, 44]. After treatment, the aforementioned enriched pathways disappeared, and the enrichment was mainly significant in the PI3K‐AKT‐mTOR signaling pathway (Figure 5h), which was consistent with the predominant enrichment of the treatment‐reversed metabolites.

**FIGURE 5:**
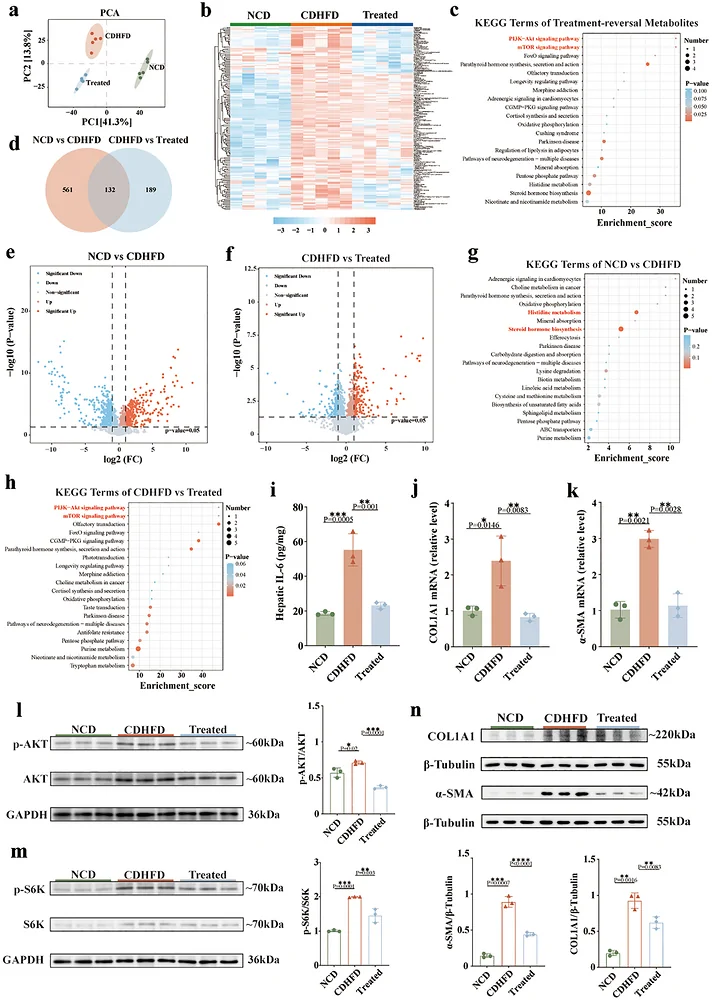
Mechanism exploration of IL22‐EcN/Gel combined with ultrasound irradiation for the treatment of MASH. (a) Different metabolomic profiles of mice from different groups visualized by principal component analysis (PCA). (b) Heatmap of treatment‐reversal metabolites from different groups. (c) KEGG pathway analysis of treatment‐reversal metabolites. (d) Venn diagram of differential metabolites in serum metabolomics analysis in the NCD group vs. CDHFD group and CDHFD group vs. treated group (IL22‐EcN/Gel + US treatment). (e, f) Volcano plot of differential metabolite expression in the NCD group vs. CDHFD group (e), CDHFD group vs. treated group (f). (g, h) KEGG pathway analysis of differential metabolites between the NCD group vs. CDHFD group (g), CDHFD group vs. treated group (h). (i) Hepatic IL‐6 levels from different groups of mice (n = 3 biologically independent samples). (j, k) mRNA expression levels of COL1A1 (j) and α‐SMA (k) determined by qRT‐PCR. (l‐n) WB of p‐AKT, AKT (l), p‐S6K, S6K (m) and COL1A1, α‐SMA (n) in liver tissues after different treatments. a‐h were n = 5 biological independent samples. P values for a‐h were calculated by Fisher's exact test. Data in i‐n were repeated three times independently. Statistical analyses for i‐n were calculated by one‐way ANOVA followed by Tukey's multiple comparison test. *p* > 0.05 (not significant, n.s.), *p < 0.05, **p < 0.01, ***p < 0.001 and ****p < 0.0001.

It has been reported that IL‐6, a classic pro‐inflammatory cytokine, acts as a critical upstream regulator of the PI3K‐AKT‐mTOR signaling pathway [45, 46]. To investigate its regulatory role in this pathway in MASH, we measured the expression levels of hepatic IL‐6 of mice from each group. The results showed that compared with the NCD group, the hepatic IL‐6 level was significantly elevated in the CDHFD group, whereas it was markedly reduced after treatment with IL22‐EcN/Gel + US (Figure 5i). These findings indicated that the treatment not only lowered portal vein LPS levels but also effectively reduced hepatic IL‐6 expression. To further elucidate the regulatory role of the PI3K‐AKT‐mTOR pathway in MASH, we subsequently evaluated the expression changes of key molecules in this pathway. Quantitative Real‐Time Reverse Transcription Polymerase Chain Reaction (qRT‐PCR) results showed the expression of fibrosis‐related genes (COL1A1 and α‐SMA) was significantly increased in the CDHFD group and markedly decreased after treatment (Figure 5j,k). These findings were further confirmed at the protein level: WB analyses revealed that the protein expression of p‐AKT, p‐S6K, COL1A1 and α‐SMA was significantly increased in the CDHFD group, while IL22‐EcN/Gel combined with US treatment significantly reduced the expression levels of these proteins (Figure 5l–n). Collectively, these data demonstrated that combined treatment of IL22‐EcN/Gel + US exerted its therapeutic effect by enabling targeted release of IL‐22 in the intestine, effectively restoring the intestinal barrier and remodeling the gut microbiota. This in turn reduced the translocation of LPS to the liver, thereby lowering hepatic IL‐6 levels. The subsequent inhibition of the pathological overactivation of the PI3K‐AKT‐mTOR signaling pathway, ultimately leading to significant improvement in MASH.

### 2.9 IL22‐EcN/Gel + US Treatment Ameliorated MASH‐associated Cognitive Impairment

A growing number of studies have shown that MASH patients face a significantly increased risk of cognitive impairment [47, 48]. Given that our treatment strategy has demonstrated significant efficacy in alleviating liver pathology, we sought to determine whether it could also ameliorate this extra‐hepatic complication, thereby achieving multi‐organ therapeutic benefits. Therefore, this study further explored the effect of IL22‐EcN/Gel combined with US treatment on MASH‐associated cognitive impairment, we employed the Morris water maze (MWM) test and novel object recognition (NOR) test, conducting multidimensional behavioral analyses to comprehensively assess the cognitive function levels of mice. The specific experimental process was illustrated in Figure 6a. During the first 4 days of training in the MWM test, the combined treatment shortened the latency to platform in MASH mice to a level comparable with that of the NCD group (Figure S14a). Figure 6b displayed the different swimming trajectories and time heatmaps of the three groups on the fifth day of the test without a platform. We observed that the treated mice showed a significant increase in the time in target quadrant (quadrant III), the times of crossing target quadrant (quadrant III) and platform (Figure S14b,c; Figure 6c), as well as a significant reduction in escape latency (Figure 6d). Therefore, the MWM test demonstrated that the IL22‐EcN/Gel combined with US treatment effectively reversed the spatial learning and memory impairments in MASH mice.

**FIGURE 6:**
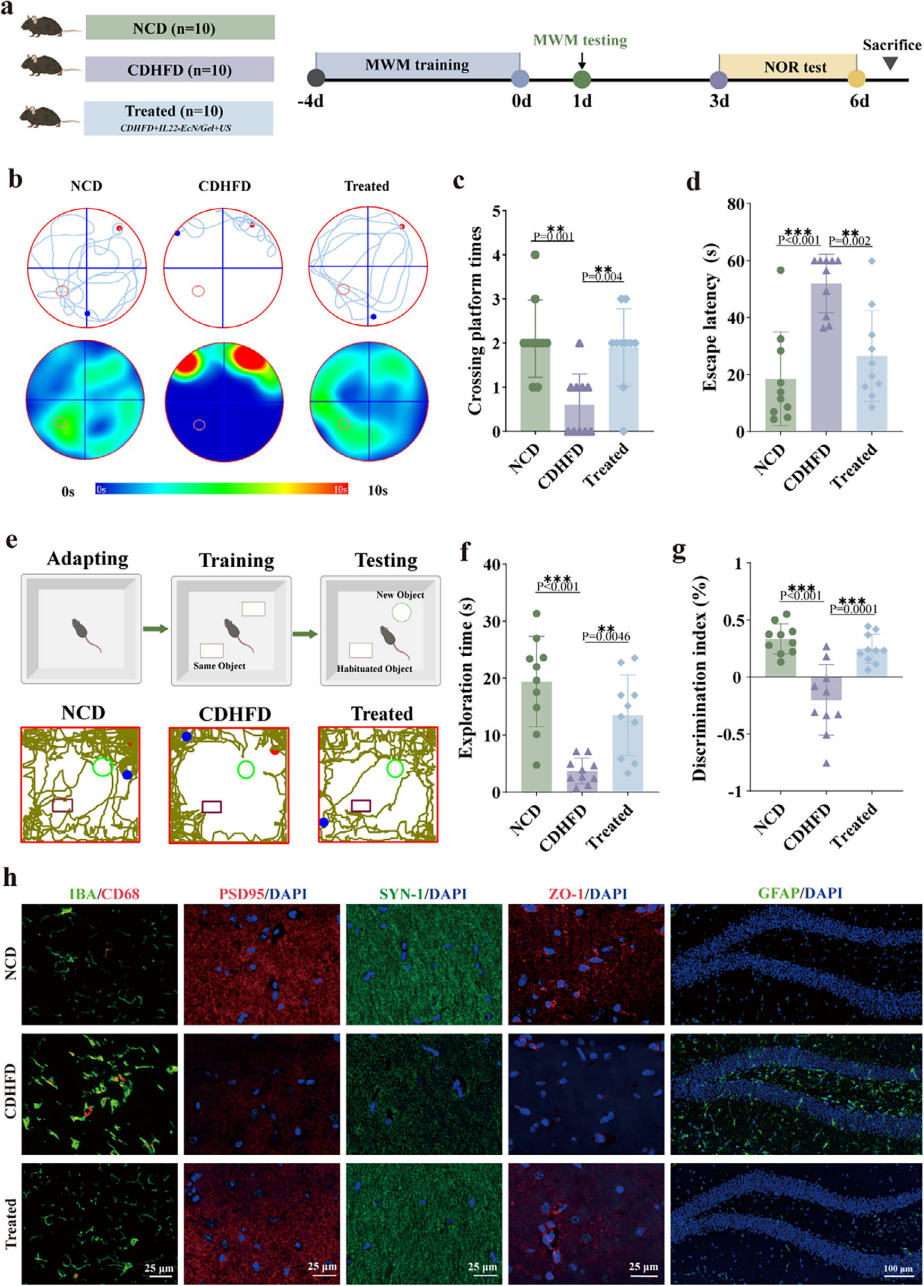
IL22‐EcN/Gel + US treatment ameliorated behavioral changes associated with MASH‐induced cognitive impairment. (a) Schematic flowchart of behavioral test procedures in mice from different experimental groups. (b) Representative swimming trajectories and time heatmaps of different groups during the testing phase of MWM. (c) The crossing number through platform of different groups of mice in the MWM test. (d) Escape latency of different groups of mice in the MWM test. (e) Representative experimental illustrations and trajectories of different groups in the NOR test. (f) The exploration time of different groups of mice in the NOR test. (g) The discrimination index of different groups of mice in the NOR test. (h) Representative immunofluorescence staining images of the mouse hippocampus. Microglia were labeled with IBA‐1 (green) and CD68 (red); astrocytes were labeled with GFAP (green); synapses were labeled with SYN‐1 (green) and PSD95 (red); the BBB was labeled with ZO‐1 (red); and cell nuclei were labeled with DAPI (blue). Data in c, d, f and g are presented as means ± SD from n = 10 biologically independent experiments. P values were calculated by one‐way ANOVA followed by Tukey's multiple comparison test. **p < 0.01 and ***p < 0.001.

In the NOR test, Figure 6e displayed the trajectories of the exploratory behavior of three groups of mice toward novel objects. Compared with the NCD group, the mice fed CDHFD exhibited significant recognition memory impairment, which was specifically manifested as reduced exploration time, discrimination index and preference index. However, after treatment with IL22‐EcN/Gel + US therapy, these cognitive deficits in MASH mice were markedly improved (Figure 6f,g; Figure S14d). The immunofluorescence staining of hippocampal tissue revealed that in CDHFD mice, the fluorescence intensity of microglial (IBA‐1, CD68) and astrocytic (GFAP) markers was increased, indicating enhanced neuroinflammatory responses. Meanwhile, the fluorescence signals of synaptic markers (SYN‐1, PSD95) and a key blood‐brain barrier (BBB) protein (ZO‐1) were reduced, suggesting impaired synaptic function and compromised BBB integrity. These indicators were reversed after combined treatment (Figure 6h). Taken together, these results confirmed that the IL22‐EcN/Gel combined with US irradiation significantly ameliorated spatial memory and object recognition memory impairments in MASH mice, demonstrating considerable potential for treating MASH‐associated cognitive dysfunction.

### 2.10 Therapeutic Effects of IL22‐EcN/Gel + US Treatment on MASH in Rats

To validate the generalizability of IL22‐EcN/Gel combined with US irradiation for treating MASH, we further evaluated the efficacy of this therapeutic strategy in a MASH rat model. Conventional B‐mode ultrasonography (Figure S15, including liver‐kidney section and the section below the right costal margin) revealed improved liver echogenicity in the treated group compared to the CDHFD group. The histopathological analysis further demonstrated that the treated group exhibited ameliorated hepatic steatosis, inflammatory infiltration and fibrosis relative to the CDHFD group (Figure S16). Subsequently, we employed the MWM test to investigate the effect of IL22‐EcN/Gel combined with US irradiation on MASH‐associated cognitive dysfunction. As expected, during the first five days of training, MASH rats showed increased latency to platform compared to the other two groups (Figure S17a). After treatment, spatial memory was significantly improved, as evidenced by increased time spent in target quadrant (quadrant III), more times of crossing target quadrant and platform, as well as significantly shortened escape latency (Figure S17b‐f). In conclusion, our study established a spatiotemporally controllable sonogenetic delivery system that targeted the gut and enabled precise release of IL‐22, ultimately achieving effective treatment of MASH and its associated cognitive impairment.

## 3 Discussion

MASLD is a global public health issue. Its more advanced subtype, MASH, features hepatic inflammation, hepatocyte ballooning and accelerated fibrosis [49]. Without timely and effective intervention, it can advance to cirrhosis and even hepatocellular carcinoma (HCC) [50, 51]. The global prevalence of MASLD is notably higher in specific disease subgroups. According to a meta‐analysis incorporating 156 studies, the global prevalence of MASLD and MASH in this population was 65% (95% CI: 62–68) and 32% (95% CI: 17–51), respectively [52, 53].

However, there are currently very few therapeutic drugs available for treating MASLD/MASH in clinical practice. Studies have found that the IL‐22 receptor (IL‐22R) deficiency exacerbated HFD‐induced metabolic disorders in mice. Notably, exogenous supplementation of IL‐22 can significantly improve insulin resistance, enhance intestinal mucosal integrity, reduce endotoxin translocation and regulate hepatic lipid metabolism [54]. However, the adverse effects of long‐term systemic IL‐22 administration have been confirmed in Phase I clinical trials, primarily manifesting as dose‐dependent cutaneous toxicity [21]. Therefore, in this study, a novel gut‐targeted sonogenetic system was designed. Developing a therapeutic strategy for the localized and precise regulation of IL‐22 expression is critical. In the field of technologies for remote and precise regulation of gene expression, both optogenetics and chemogenetics, despite their widespread application, exhibit inherent limitations. Optogenetics suffers from poor tissue penetration, making it challenging to non‐invasively target deep‐seated organs [30]. Chemogenetics, due to the systemic distribution and slow pharmacokinetics of small‐molecule inducers, is often accompanied by off‐target effects and low temporal resolution, potentially introducing additional pharmacological complexities [55]. In contrast, sonogenetics has garnered significant attention for its non‐invasiveness, excellent deep‐tissue penetration capability and favorable safety profile, demonstrating considerable advantages in regulated cell therapies in recent years.

For example, Liu et al. developed a remotely controllable “ultrasonic genetic switch” system, EchoBack‐CAR T cells, which utilized a heat shock promoter (Hsp) to achieve sustained CAR expression triggered by short‐duration focused ultrasound (FUS), effectively killing tumors while avoiding off‐target toxicity [56]. In this study, the engineered probiotic employed a homologous regulation strategy: a temperature‐sensitive transcriptional switch mediated by the heat‐shock promoter Hsh enabled precise induction of IL‐22 expression under the ultrasonic thermal effects at 42°C. To regulate temperature stability and avoid tissue thermal damage, systematic acoustic parameter screening was conducted, ultimately identifying a 1:1 pulse mode (1s on, 1s off) at 0.5 W/cm^2^ intensity with continuous irradiation for 30 min as the optimal protocol. This approach efficiently activated the heat‐shock promoter while providing a safe and controllable technical foundation for gut‐targeted delivery of IL‐22. Furthermore, the inulin hydrogel encapsulated on the surface of the engineered probiotic enhanced adhesion, ensuring sustained colonization in the colon. This encapsulation strategy not only promoted the proliferation of the engineered probiotic but also improved oral bioavailability, thereby extending the therapeutic effect. It is particularly important to emphasize that in vivo biosafety is a prerequisite for the clinical translation of this sonogenetic system. A 30‐day systematic safety evaluation demonstrated that activation with the established ultrasound parameters (0.5 W/cm^2^, 1s on/1s off) in healthy mice treated with IL22‐EcN/Gel did not cause significant histopathological damage or adverse reactions.

The core advantage of the novel sonogenetic system targeting the gut designed in this study lies in its use of exogenous ultrasound as a non‐invasive, tissue‐penetrating remote switch, enabling spatiotemporally precise regulation of IL‐22 expression within the intestinal tract. This represents a fundamental breakthrough compared to traditional IL‐22 administration or conventional engineered bacterial therapies. It allows for on‐demand, timed and quantitative control over the release of the therapeutic protein (IL‐22) in the gut, thereby avoiding the off‐target risks associated with systemic exposure. In vivo safety evaluations confirmed that this sonogenetic system exhibited excellent biosafety, significantly broadening the therapeutic window of IL‐22‐based treatment.

In the CDHFD‐induced MASH model constructed in this study, the combined treatment of IL22‐EcN/Gel and ultrasound demonstrated multi‐dimensional therapeutic advantages. First, the IL22‐EcN/Gel+US group significantly inhibited weight gain in MASH mice, which appeared directly related to reduced food intake and may be closely related to the appetite regulation mediated by the specific upregulation of appetite controlling peptide YY (PYY) by IL‐22 [52]. Second, the IL22‐EcN/Gel+US group showed marked improvements in hepatic steatosis, inflammation, fibrosis and oxidative stress damage, with efficacy significantly superior to other single or combined treatment groups. It is worth noting that this combination therapy also effectively restored the expression of key intestinal barrier proteins (Claudin 3, Occludin and E‐Cadherin) and achieved gut homeostasis by remodeling gut microbiota and reducing LPS translocation. It is known that when LPS translocate through the impaired intestinal barrier into the portal circulation and hippocampus, it triggers excessive production of pro‐inflammatory cytokines (such as IL‐6, IL‐1β and TNF‐α) in the liver [57]. Furthermore, this combined treatment also inhibited astrocyte activation and neuroinflammation, while restoring BBB function. Ultimately, it was observed to ameliorate relevant cognitive impairment in the MASH model.

Finally, this study elucidated the mechanism by which this novel sonogenetic system reverses MASH. Based on the KEGG analysis of serum metabolomics, the metabolic alterations in the combined treatment group (IL22‐EcN/Gel+US) were significantly enriched in the PI3K‐AKT‐mTOR signaling pathway. Key molecules associated with this pathway included AKT, p‐AKT, S6K, p‐S6K, COL1A1 and α‐SMA. We confirmed that the combined therapy markedly ameliorated core pathological features of MASH (steatosis, inflammation, and fibrosis). This therapeutic effect was achieved by reducing the LPS translocation, which in turn lowered hepatic IL‐6 levels and consequently inhibiting the pathological overactivation of the PI3K‐AKT‐mTOR signaling pathway. In our gut‐restricted delivery model, the inhibition of this pathway is primarily driven by an IL‐6‐dependent indirect gut‐liver axis mechanism, initiated by reduced portal LPS leakage. Nevertheless, as reported in systemic administration models [58], a direct modulatory effect of any gut‐derived IL‐22 that reaches the liver on this pathway cannot be excluded. These two regulatory modes are complementary, and our focus on hepatic IL‐6 was intended to track inter‐organ inflammatory signaling, rather than to imply that IL‐22 acts solely through IL‐6 mediation.

In our study, although ultrasound effectively induces IL‐22 expression and release, the relatively long exposure time required by the current protocol remains a barrier to clinical translation. To address this limitation, several optimization strategies could be considered in future studies.  First, acoustic signal amplification via cavitation nuclei—using FDA‐approved contrast agents or acoustically responsive nanomaterials—could enhance mechanotransduction efficiency, potentially reducing exposure time to under 5 min without compromising IL‐22 yield. Second, fine‐tuning pulse parameters (e.g., higher peak negative pressure with a lower duty cycle) could improve transient bacterial activation while maintaining thermal safety within FDA limits. Third, integration with emerging flexible, wearable ultrasound patches [59] would enable hands‐free therapy, rendering prolonged regimens more acceptable for chronic care. Collectively, these physics‐ and engineering‐driven approaches offer a clear roadmap for reducing treatment time and facilitating clinical translation.

In conclusion, as shown in Figure 7, this study pioneered the integration of sonogenetic technology and probiotic therapy for the treatment of MASH, establishing a novel combined treatment paradigm that enabled spatiotemporally precise control of engineered bacteria by ultrasound. By targeting and regulating the gut‐liver axis, this strategy provided a breakthrough approach for the safe and precise intervention in MASH.

**FIGURE 7:**
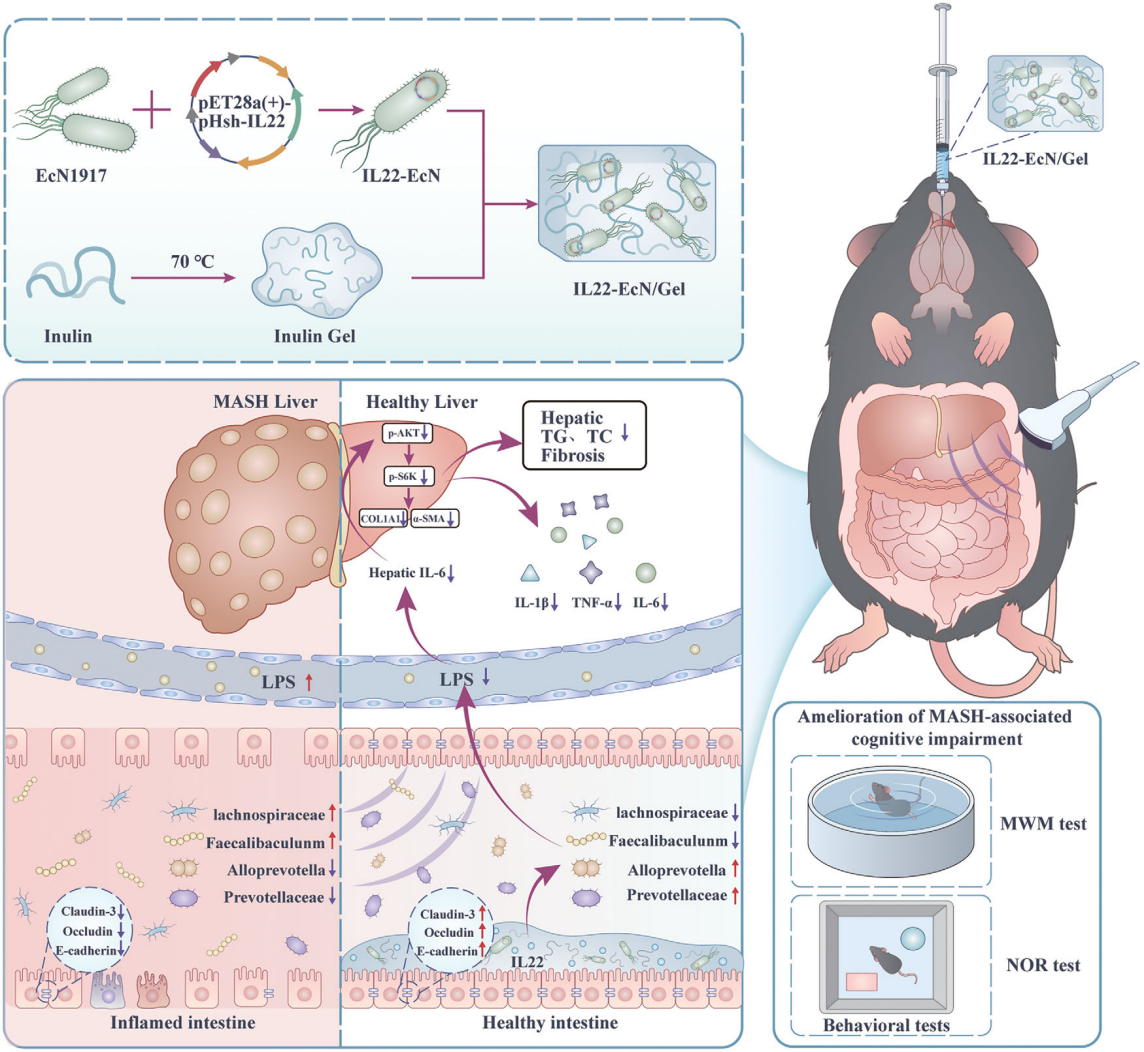
Overview schematic of engineered probiotics based on spatiotemporally precise ultrasound manipulation treat MASH and its cognitive impairment via regulating the gut‐liver axis. We successfully constructed the engineered probiotic (IL22‐EcN) and encapsulated it using heating‐cooling process to form an inulin hydrogel‐based IL22‐EcN/Gel system. After oral administration to MASH model mice, under ultrasound regulation, this sonogenetic probiotic system achieved precise intestinal targeting and controlled release of IL‐22. This process remodeled the gut microbiota, improved intestinal barrier function, and further reduced LPS levels in the portal vein, leading to decreasing hepatic IL‐6 production. Ultimately, by inhibiting the pathological overactivation of the PI3K‐AKT‐mTOR signaling pathway in the liver, these changes led to significant alleviation of MASH and associated cognitive impairment.

## 4 Materials and Methods

### 4.1 Mice

Male C57BL/6 mice (seven weeks old, approximately 22 g) were sourced from Shanghai Ji Hui Experimental Animal Co., Ltd. All animal studies received ethical approval from the Animal Ethics Committee of Shanghai Tenth People's Hospital affiliated to Tongji University (SHDSYY‐2024‐2024250166). The mice were housed under specific pathogen‐free (SPF) conditions (temperature: 20–22°C; humidity: 45 ± 5%; 12‐h light/dark cycle) with free access to food (specific diet as described below) and water.

### 4.2 Animal Experiments

Following a one‐week acclimation, the mice were subjected to experimental feeding with either a standard diet (TP23522, Trophic Animal Feed High‐tech Co., Ltd) or a CDHFD (TP3630076A, Trophic Animal Feed High‐tech Co., Ltd).

After 16 weeks of CDHFD feeding, the first mouse model was successfully established. A total of 35 mice were randomly divided into 7 groups (n = 5 per group): I) NCD, II) CDHFD, III) CDHFD + US irradiation, IV) CDHFD + EcN, V) CDHFD + IL22‐EcN, VI) CDHFD + IL22‐EcN/Gel, VII) CDHFD + IL22‐EcN/Gel + US irradiation. Mice in the NCD, CDHFD and CDHFD + US irradiation groups received oral gavage of 200 µL PBS every two days. Mice in the remaining groups were gavaged every two days with the corresponding bacterial suspension (200 µL, EcN or IL22‐EcN = 1×10^8^ CFU, inulin gel = 0.7 g/mL).

Mice in the CDHFD + US irradiation group and the CDHFD + IL22‐EcN/Gel + US irradiation group underwent 30 min ultrasound irradiation (0.5 W/cm^2^, 1s on/1s off) applied to the abdominal area 12 h after each gavage session every two days. Mice body weight was recorded at weekly intervals. After 8 weeks, fecal collection for 16S rRNA sequencing was performed. The mice that fasted overnight were euthanized. Blood was collected via orbital bleeding, and liver, spleen, kidney and distal colon tissues were harvested for subsequent analysis.

After 10 weeks of HFHC feeding, the second mouse model was successfully established. A total of 20 mice were randomly divided into 4 groups (n = 5 per group): I) NCD, II) HFHC, III) HFHC + IL22‐EcN/Gel, IV) CDHFD + IL22‐EcN/Gel + US irradiation. Mice in the NCD and HFHC groups received oral gavage of 200 µL PBS every two days. Mice in the HFHC + IL22‐EcN/Gel group were gavaged every two days with the corresponding bacterial suspension (200 µL, IL22‐EcN = 1×10^8^ CFU, inulin gel = 0.7 g/mL). Mice in the HFHC + IL22‐EcN/Gel + US irradiation group underwent 30 min ultrasound irradiation (0.5 W/cm^2^, 1s on/1s off) applied to the abdominal area 12 h after each gavage session every two days. Mice body weight was recorded at weekly intervals. After 8 weeks, the mice that fasted overnight were euthanized. Blood was collected via orbital bleeding, and relevant tissues were harvested for subsequent analysis.

### 4.3 Bacteria Strains

We purchased *E. coli* Nissle 1917 (EcN1917) glycerol bacteria from Ningbo Mingzhou Biotechnology Co., Ltd. and EcN‐Lux glycerol bacteria from Fenghai Biotechnology Co., Ltd. The strains were cultured overnight at 37°C with 200 rpm shaking in 5 mL of Luria‐Bertani (LB) liquid medium. The plasmids pET28a (+)‐pHsh‐IL22 and pET28a (+)‐pHsh‐Lux were both obtained from Ningbo Mingzhou Biotechnology Co., Ltd. These plasmids were respectively transfected into EcN1917 to generate IL22‐EcN and Lux‐EcN. Additionally, pET28a (+)‐pHsh‐IL22 was transfected into EcN‐Lux to construct IL22‐EcN‐Lux. All engineered strains were cultured in kanamycin‐supplemented LB liquid medium (50 µg/mL).

### 4.4 Construction of Plasmids

Using pUC57‐kan‐pSH‐IL‐22 as a template with primers HSH‐28A.F/R, the fragment pHsh‐IL‐22 (886 bp) was amplified. Using pET28a (+) as a template with primers 28A‐HSH.F/R, the vector fragment pET28a (+) (3539 bp) was amplified. The two fragments were assembled using seamless cloning technique. Transformation of the ligation product into *E. coli* DH5α was followed by selection of transformants on LB‐kanamycin plates. The successfully constructed plasmid, verified by PCR and sequencing, was designated as pET28a (+)‐pHsh‐IL22.

Using the plasmid ilux pGEX (‐) as a template and 1LXC‐28PSH.F/1LXC‐LXA.R as primers, the first part of the LuxCDABE fragment (2488 bp) was amplified; 2LXA‐LXE.F/2LXA‐LXE.R were primers, amplifying the second part of the LuxCDABE fragment (2314 bp). 3LXE‐28PSH.R/3LXE‐LXA.F were primers, amplifying the third part (1159 bp) of the LuxCDABE fragment (1159 bp). Using the plasmid pET28a(+)‐pHsh‐IL22 as the template and 28PSH‐LUX.R/28PSH‐LUX.F as the plasmid, the expression vector pET28a (+) ‐pHsh fragment (3816 bp) was amplified. The amplification fragments of the above three parts and the four fragments of the vector pE28a (+) ‐pHsh were ligated by seamless cloning technology. The ligation product was transformed into E. coli DH5α and plated on LB plates supplemented with kanamycin for selection of positive clones. After verification by PCR and sequencing, the constructed vector was named pET28a (+) ‐pHsh‐Lux.

All plasmids, strains and primers used in this study were listed in Table S1, Table S2 and Table S3, respectively.

### 4.5 Construction of Strains

The overnight cultured EcN1917 was transferred to sterile fresh LB liquid medium at a 1:100 dilution and incubated at 37°C with shaking (200 rpm) until OD_600_ reached approximately 0.8. The bacterial suspension was then placed on ice for 15 min. Wash the bacteria three times with 10 mL of pre‐cooled sterile 10% glycerol and discard the supernatant. Collect the precipitate into sterile EP tubes, resuspend it on ice in an appropriate amount of pre‐cooled sterile 10% glycerol, mix well and aliquot, 100 µL per tube. Take 5 µL of the pre‐cooled pET28a (+) ‐pHsh‐IL22 plasmid solution (containing approximately 1 µg of the plasmid) and thoroughly mix it with the above‐mentioned competent bacterial suspension. After mixing, the mixture was kept on ice for 15 min, transferred into a pre‐cooled 2 mm electroporation cup for 2.5 kV electroporation. Immediately after electroporation, 900 µL of LB liquid medium (37°C) was added. The bacterial liquid was placed at 37°C, resuscitated at 200 rpm for 1 h, and then centrifuged at 8000 rpm for 5 min. After discarding 900 µL of the supernatant, the remaining bacteria was resuspended and transferred into kanamycin‐supplemented (50 µg/mL) LB liquid medium for overnight incubation at 37°C, yielding the engineered IL22‐EcN strain.

The 5 µL pre‐cooled pET28a (+) ‐pHsh‐Lux plasmid solution was thoroughly mixed with the prepared competent EcN1917 suspension. The Lux‐EcN strain was subsequently obtained using the same electroporation and culture procedure as described above.

The 5 µL pre‐cooled of pET28a (+)‐pHsh‐IL22 plasmid solution was added to the prepared electrocompetent EcN‐Lux suspension. The IL22‐EcN‐Lux strain was subsequently obtained following the identical electroporation and culture procedure.

### 4.6 IL22‐ EcN Culture

Following overnight culture in LB medium with kanamycin (50 µg/mL) at 37°C with shaking, the IL22‐EcN suspension was divided into a sonicated group and a non‐sonicated control group. The sonicated group received 30 min of ultrasound irradiation (0.5 W/cm^2^, 1s on/1s off). The next day, the optical density (OD_600_) of both cultures was measured, and each was diluted to an OD_600_ of 0.25. The diluted bacterial suspensions were then allowed to grow until the logarithmic phase was reached. At each time point, samples were taken from both groups, and their optical density was determined with a Nanodrop 2000c spectrophotometer (Thermo Scientific) in cuvette mode.

### 4.7 DMAO/PI Staining

In accordance with the manufacturer's protocol, the bacterial viability was determined using the Live/Dead Bacterial Staining Kit (DMAO/PI) (Beyotime, C2030S). First, IL22‐EcN was grown in LB liquid medium to the logarithmic phase, and an appropriate amount of bacterial culture was taken and adjusted to an OD_600_ of 0.25. Bacterial culture was incubated with the freshly prepared Live/Dead staining working solution at a 100:1 ratio. After 15 min of incubation at 37°C in the dark, the staining results were visualized with a fluorescence microscope.

### 4.8 Synthesis of IL22‐EcN/Gel

To prepare the probiotic hydrogel, an inulin solution (0.7 g/mL) was first vortexed and then heated to 70°C for 10–15 min in a water bath. After natural cooling to 37°C, IL22‐EcN was mixed into the inulin solution, followed by incubation at room temperature for 12 h to form IL22‐EcN/Gel.

### 4.9 Cell/Bacterial Viability Assay

The viability of various cell lines (IEC, HCT‐8, AML‐12, HK‐2, HSF) and IL22‐EcN, with or without US irradiation, was assessed using an Enhanced CCK‐8 kit (Beyotime Biotechnology, C0042). To this end, cells were seeded in 60 mm culture dishes prior to the assay. Cells or IL22‐EcN suspensions were inoculated in 96‐well cell culture plates, with 2×10^3^ cells per well/100 µL, and a blank group and a control group were set up. The experimental groups were exposed to US irradiation (0.5 W/cm^2^, 1s on/1s off) for 30 min. After adding 10 µL of enhanced CCK‐8 solution per well and thorough mixing, the plate was incubated at 37 °C for 1–4 h, and the optical density (OD) at 450 nm was measured with a microplate reader (Bio‐Tek ELx800, USA) for viability assessment.

### 4.10 In Vitro Degradation Experiment of IL22‐EcN/Gel

The empty EP tube was weighed and recorded as m_0_. Then, 1 mL of the mixed IL22‐EcN and inulin solution was aliquoted into the EP tube. After gelation, the total mass was measured and recorded as m_1_. The IL22‐EcN/Gel was incubated in SGF (pH = 1.5; Solarbio, A7921) and SIF (pH = 6.8; Solarbio, A1791) separately at room temperature. At predetermined time points, the supernatant was removed, and the remaining gel together with the EP tube was weighed (recorded as m). The percentage of mass loss was calculated using the formula: (m—m_0_) / (m_1_ – m_0_) × 100%.

### 4.11 In Vitro Residence and Release Assay of IL22‐EcN/Gel

Equal concentrations of IL22‐EcN/Gel or IL22‐EcN was resuspended in SGF (pH = 1.5; Solarbio, A7921) or SIF (pH = 6.8; Solarbio, A1791), respectively, and cultured in a shaking incubator at 37 °C to mimic the gastrointestinal environment. At predetermined time points, bacterial suspensions were collected, washed three times with PBS, and the pellets were resuspended. After serial dilution in PBS, the suspensions were plated on kanamycin‐LB plates and incubated overnight at 37°C for colony counting.

### 4.12 In Vivo Gastrointestinal Colonization of IL22‐EcN/Gel

To explore the colonization efficiency of IL22‐EcN/Gel in the gastrointestinal tract, healthy male C57BL/6 mice were randomly assigned and orally gavage with either IL22‐EcN/Gel or IL22‐EcN bacterial suspension at a dose of 1×10^8^ CFUs. Fresh fecal samples were collected daily for 7 consecutive days, weighed, and immediately homogenized in ice‐cold PBS. Serial dilutions of homogenized fecal suspensions were plated onto kanamycin‐LB plates and incubated overnight at 37 °C. Viable bacteria were quantified by counting bacterial colonies.

### 4.13 Evaluation of Intestinal Retention In Vivo

The MASH mice (the CDHFD‐induced model constructed in this study) were randomly assigned to two groups (n = 3). After oral gavage with IL22‐EcN‐Lux and IL22‐EcN‐Lux/Gel (200 µL, IL22‐EcN‐Lux = 1×10^8^ CFU, inulin gel = 0.7 g/mL), the MASH mice were anesthetized with 2% isoflurane at 4 h, 8 h, 12 h, 24 h, 48 and 72 h respectively. The bioluminescence images were observed using the VISQUE system.

### 4.14 In Vivo Safety Assessment

The 7‐week‐old male C57BL/6 mice were randomly assigned to four groups (n = 3) after one week of acclimatization in the animal facility. After gavage of IL22‐EcN/Gel (200 µL, IL22‐EcN = 1×10^8^ CFU, inulin gel = 0.7 g/mL), the abdominal area of the mice was exposed to 30 min of ultrasound irradiation (0.5 W/cm^2^, 1s on/1s off). Orbital blood sampling was collected on the third, seventh and 30th days. The collected blood was clotted for 2 h and then centrifuged (3000 rpm, 4 °C, 20 min) to obtain serum for assessing liver and kidney function. Whole blood samples were used for biochemical analysis. For H&E staining, major organs (heart, liver, spleen, lung, kidney, intestine and brain) were collected.

### 4.15 Protein Extraction and Western Blot

Following denaturation in 5X SDS‐PAGE loading buffer (100°C, 5 min), the collected protein samples were separated via 10% SDS‐PAGE gel electrophoresis and then transferred to PVDF membranes. Incubate the sample with the rapid blocking buffer at room temperature for 15 min, followed by three washes with TBST (TBS containing 0.1% Tween‐20), each lasting 5 min. The membranes were then incubated overnight at 4 °C with a primary antibody solution. After subsequent TBST washes, a 1‐h incubation at room temperature with a secondary antibody (HRP Goat Anti‐Rabbit IgG (H+L), AS014) diluted in TBST was performed. Following a final series of TBST washes, and the target proteins were visualized with Pierce ECL Western Blotting Substrate.

### 4.16 Cytokine Detection

After treating CDHFD mice in different groups for 8 weeks, orbital blood was collected from mice that had fasted overnight and the serum was obtained by centrifugation. Serum levels of IL‐22, IL‐6, IL‐1β and TNF‐α were determined with specific enzyme‐linked immunosorbent assay (ELISA) kits following the manufacturers’ protocol.

### 4.17 Histological Analysis

For histological analysis liver tissues were collected from all groups after 8 weeks of treatment, followed by weighing and photographing, and paraformaldehyde fixation. H&E, Oil Red O, and Masson staining were performed to evaluate hepatic inflammation, lipid droplet infiltration, and the extent of liver fibrosis, respectively.

### 4.18 qRT‐PCR

Total RNA was extracted from liver tissues of mice in the NCD, CDHFD and treated groups using TRIzol reagent (Thermo Fisher Scientific). RNA concentration and purity were measured using a Nanodrop 2000 spectrophotometer (Thermo Fisher Scientific). Reverse transcription products (cDNA) were obtained using the reverse transcription kit (Thermo Fisher Scientific, K1691), and amplification was finally completed on a fluorescence quantitative PCR instrument (ABI Step one plus, Applied Biosystems). The expression level of target mRNAs was normalized using GAPDH as the internal reference. Primer sequences for COL1A1 and α‐SMA were provided in Table S4.

### 4.19 FITC‐dextran Permeability Assay

Mice were fasted for 4–6 h and then orally administered 200 µL of 4 kDa FITC‐Dextran (600 mg/kg; Beyotime, ST2930) by gavage. At 4 h after gavage, blood was collected and serum was separated. The fluorescence intensity of FITC‐Dextran in the serum was measured using fluorescence spectrophotometry with excitation at 490 nm and emission at 520 nm, and the concentration was calculated by means of a standard curve.

### 4.20 LPS Detection

After anesthesia, portal vein blood was collected from mice in the NCD, CDHFD and treated groups, and the serum was separated. The serum LPS concentration in each group was determined by the LPS ELISA kit (Cusabio, CSB‐E13066m), adhering strictly to the manufacturer's protocol.

### 4.21 Assessment of Intestinal Epithelial Barrier Function

After 8 weeks of treatment, distal colon tissues of mice in each group were collected and rapidly placed in electron microscopy fixative, and stored at 4 °C for subsequent TEM observation of tight junctions, adherens junctions and microvilli morphology. Distal colon cryosections were immunostained to evaluate intestinal barrier function. Briefly, sections fixed in ice‐cold acetone were blocked (1% BSA + avidin/biotin) and incubated with primary antibodies against E‐cadherin (14472, Cell Signaling Technology), Claudin‐3 (83609, Cell Signaling Technology), and Occludin (91131, Cell Signaling Technology) at 4°C overnight. After applying fluorescent secondary antibodies for 1 h protected from light, visualization was conducted using a laser scanning confocal microscope (TCS SP8, Leica).

### 4.22 16S rRNA Gene Sequencing Analysis

After 8 weeks of treatment, fecal samples were collected and preserved in liquid nitrogen. Bacterial genomic DNA was extracted from these samples using the DNeasy PowerSoil Kit (Qiagen, Hilden, Germany), with its quality verified by a NanoDrop 2000 spectrophotometer (Thermo Fisher Scientific, Waltham, MA, USA) and agarose gel electrophoresis. The V3–V4 hypervariable regions were PCR‐amplified (343F: 5′‐ tacggraggcagag ‐3′; 798 r: 5“‐AGGGTATCTAATCCT‐3”) in a 25 µL reaction system. The quality of the amplification products was verified by gel electrophoresis, purified with Agencourt AMPure XP beads (Beckman Coulter Co., USA) and quantified by Qubit dsDNA assay kit. After adjusting concentration, paired‐end 250 bp sequencing was performed using the Illumina NovaSeq 6000 system (Illumina Inc., San Diego, CA, USA) provided by Novogene (OE Biotech Company, Shanghai, China). Bioinformatic processing involved quality filtering, denoising, and Amplicon Sequence Variant (ASV) clustering in QIIME 2, followed by SILVA‐based taxonomy annotation (Release 138).  Unweighted Unifrac‐based principal coordinate analysis (PCoA) was further conducted utilizing QIIME 2.

### 4.23 Metabolomic Analysis

Metabolomic analysis of mouse serum or fecal samples (n = 5 per group) was performed based on the Waters ACQUITY UPLC I‐Class Plus/ Thermo QE HF system (Waters/Thermo, USA). The extracted metabolite data were processed by RSD filtering, score‐based filtering and log2 transformation. Principal component analysis (PCA) was applied for multidimensional data analysis, while volcano plots and cluster heatmaps were used to screen differentially abundant metabolites. Differential metabolites were mapped to metabolic pathways for enrichment analysis using the KEGG database, and the results were visualized using KEGG bubble plots.

### 4.24 Morris Water Maze (MWM) Test

Spatial learning and memory were assessed using the Morris water maze (MWM) test. The maze consisted of a circular pool (diameter 1.5‐2 m, depth 50 cm) filled with opaque, temperature‐controlled water (22‐24 °C) water and divided into four equal quadrants. A hidden platform (diameter 10 cm) was positioned in the target quadrant (quadrant III in this study), with its surface slightly submerged to allow the mice/rats to rest on it. Distinct visual cues were placed around the pool to aid the animals in forming spatial memory. A digital camera, equipped with video recording and tracking capabilities, was mounted directly above the center of the pool to document swimming paths and quantify escape latency. Each test group included 10 mice or 6 rats.

During the consecutive 4‐day training period for mice (5 days for rats), the trained mice swam four times a day to find hidden platforms, with adequate inter‐trial intervals to prevent excessive fatigue. The escape latency was quantified for each trial, starting from four different release points corresponding to the four quadrants. If mice unable to locate the platform within 60 s was gently assisted to the location and allowed to stay for 10 s. The escape latency for such trials was scored as 60 s. Upon removal of the platform on day 5 (day 6 for rats), a probe trial was conducted. Mice, released from the quadrant opposite to the target quadrant (quadrant III), swam freely while their time in the target quadrant and the numbers of crossing platform were quantified.

### 4.25 Novel Object Recognition (NOR) Test

In the novel object recognition (NOR) test, which evaluates learning and memory, mice first underwent habituation on day one. This involved a 5‐min free exploration session in an empty chamber (60 cm × 60 cm × 40 cm) for environmental acclimation. On the second day, two identical rectangular objects were positioned in the upper‐right and lower‐left corners of the chamber and then put the mouse in and let it freely explore for 5 min. The formal test was conducted on the third day, during which the rectangular object in the upper‐right corner was replaced with a circular one. Each mouse then underwent a 5‐min recognition trial in the chamber, and the exploration time directed toward each of the two different objects was recorded. Between trials, the chamber was thoroughly cleaned to remove any waste and sanitized with 75% ethanol to eliminate the odor. Schematic overview of animal allocation and downstream use of biological samples following behavioral assessments was provided in Figure S18.

### 4.26 Immunofluorescence Staining of Hippocampus

To comprehensively evaluate neuroinflammation, the integrity of BBB and synaptic function, immunofluorescence staining was performed. Fixed brain sections (20 µm thick) were blocked with 5% BSA for 30 min. They were then incubated with primary antibodies against IBA‐1, CD68, ZO‐1, GFAP, SYN‐1 and PSD‐95 overnight at 4 °C. Following three PBS washes, the sections were treated with appropriate secondary antibodies for 1 h. Imaging analysis was finally carried out using a laser scanning confocal microscope (TCS SP8, Leica).

### 4.27 Statistical Analysis

Statistical analysis was conducted with GraphPad Prism (version 9.0.0). Data are expressed as mean ± standard deviation (SD). For comparisons between two groups, a two‐tailed unpaired Student's t‐test was applied. For comparisons among more than two groups, one‐way or two‐way analysis of variance (ANOVA) was applied. Statistical significance was defined as follows: *p* > 0.05 (not significant, n.s.), *p < 0.05, **p < 0.01, ***p < 0.001 and ****p < 0.0001. Quantitative analysis of WB results was conducted using ImageJ software (version 1.8.0).

## Author Contributions

**Chunya Ji**: conceptualization, data curation, formal analysis, writing – original draft, writing – review and editing, funding acquisition, supervision, resources, project administration, methodology. **Yan Fang**: methodology, validation, investigation, writing – review and editing. **Shen Zhang**: methodology, validation, investigation, writing – review and editing. **Weichen Xu**: investigation. **Wenyun Guo**: investigation. **Jing Chen**: investigation. **Mingrui Zhu**: investigation. **Congjian Wen**: investigation. **Siming Chen**: investigation. **Yuli Zhu**: supervision, funding acquisition, project administration, resources. **Huixiong Xu**: conceptualization, supervision, project administration, resources, writing – review and editing, funding acquisition. **Haohao Yin**: conceptualization, supervision, project administration, funding acquisition, resources, writing – review and editing.

## Funding

This work was funded by the National Nature Science Foundation of China (Grant Nos. 82430064, 81927801, 82302206 and 82102050), Scientific Research and Development, Fund of Zhongshan Hospital of Fudan University (Grant Nos. 2022ZSQD07), Shanghai Sailing Program (Grant Nos. 23YF1441600), China Postdoctoral Science Foundation (Grant Nos. 2023TQ0073), Health Commission Health Sector Clinical Research Special Clinical Project (Grant Nos. 202340087) and Suzhou Gusu Health Talents Program (Grant Nos. GSWS2023027).

## Consent Statement

No written consent has been obtained from the patients as there is no patient identifiable data included in this series.

## Conflicts of Interest

Authors declare that they have no conflicts of interest.

## Supporting information




**Supporting File**: advs78100‐sup‐0001‐SuppMat.docx.

## Data Availability

All data are available in the main text and/or the Supplementary Materials.
