# Supplementary material for: Sonogenetic Control of Engineered Probiotics for the Treatment of MASH by Modulating the Gut‐Liver Axis
Source: Adv Sci (Weinh). 2026 Oct 8:e78100. Online ahead of print. doi: 10.1002/advs.78100 (PMC13650197; doi:10.1002/advs.78100)
Supplement: Supplementary file 1 [file ADVS-9999-e78100-s001.docx]

**Sonogenetic control of engineered probiotics for the treatment of MASH by modulating the gut-liver axis**

**Chunya Ji****^1#^, Yan Fang^1#^, Shen Zhang^1, 2, 3#^, Weichen Xu^2^, Wenyun Guo^1^, Jing Chen^1^, Mingrui Zhu^1^, Congjian Wen^1^, Siming Chen^4^, Yuli Zhu^1^*, Huixiong Xu^1^* & Haohao Yin^1^***

^1^Department of Ultrasound, Institute of Ultrasound in Medicine and Engineering, Zhongshan Hospital, Fudan University, Shanghai 200032, China

^2^Department of Medical Ultrasound, Center of Minimally Invasive Treatment for Tumor, Shanghai Tenth People's Hospital, School of Medicine, Tongji University, Shanghai 200072, China

^3^Shanghai Engineering Research Center of Ultrasound Diagnosis and Treatment, Shanghai 200072, China

^4^School of Data Science, Fudan University, Shanghai 200433, China

^#^These authors contributed equally to this work

**Corresponding authors:**

Yuli Zhu

Department of Ultrasound, Institute of Ultrasound in Medicine and Engineering, Zhongshan Hospital, Fudan University, Shanghai 200032, China

E-mail: [zhu.yuli@zs-hospital.sh.cn](mailto:zhu.yuli@zs-hospital.sh.cn)

Huixiong Xu

Department of Ultrasound, Institute of Ultrasound in Medicine and Engineering, Zhongshan Hospital, Fudan University, Shanghai 200032, China

E-mail: [xu.huixiong@zs-hospital.sh.cn](mailto:xu.huixiong@zs-hospital.sh.cn)

Haohao Yin

Department of Ultrasound, Institute of Ultrasound in Medicine and Engineering, Zhongshan Hospital, Fudan University, Shanghai 200032, China

E-mail: [yin.haohao@zs-hospital.sh.cn](mailto:yin.haohao@zs-hospital.sh.cn)

**Fig. S1. Schematic diagram of the thermosensitive regulatory circuit in Lux-EcN.**

**Fig. S2. The effects of ultrasound irradiation on IL22-EcN and various tissue cells.**

**a** Survival rate analysis of IL22-EcN with or without US irradiation by CCK8. Data are presented as means ± SD (n=5 biologically independent samples). **b** Growth curves of IL22-EcN with or without US irradiation. Data are presented as means ± SD (n=3 biologically independent samples). **c** Representative fluorescence microscope images showing bacterial viability assessed by the DMAO/PI assay. **d and e** Cell survival analysis of normal tissue cells and tumor cells with or without US irradiation by CCK8. Data are presented as means ± SD (n=5 biologically independent samples). P value was calculated by two-tailed unpaired Student’s t-test **(a, b, d and e)**. *p* > 0.05 (not significant, n.s.)

**Fig. S3. The biodistribution of IL22-EcN-Lux and IL22-EcN-Lux/Gel in the major organs of mice after oral gavage.**

**a** Bioluminescence images of the entire gastrointestinal tract 12 hours after oral gavage with IL22-EcN-Lux and IL22-EcN-Lux/Gel. **b** Quantitative analysis of bioluminescence signal intensity in the entire gastrointestinal tract 12 hours after oral gavage. **c** Bioluminescence images of extraintestinal organs (1: brain, 2: kidney, 3: heart, 4: lung, 5: spleen, 6: liver) 72 hours after oral gavage with IL22-EcN-Lux and IL22-EcN-Lux/Gel. Data are presented as means ± SD (n=3 biologically independent samples). P value was calculated by two-tailed unpaired Student’s t-test **(b)**. ***p* < 0.01.

**Fig. S4. Infrared thermography of CDHFD-fed mice under ultrasound irradiation.**

**Fig. S5. Ultrasound-induced transgene expression via a sonogenetic system in CDHFD mice**

**a** Schematic diagram of ultrasound-mediated transgene expression via a sonogenetic system in CDHFD mice. **b** Representative bioluminescence images of time-dependent transgene expression of sonogenetic systems in CDHFD mice. **c** Quantitative analysis of bioluminescence signal intensity for time-dependent transgene expression controlled by a sonogenetic system in CDHFD mice. Data are presented as means ± SD (n=3 biologically independent samples). P values were calculated by one-way ANOVA followed by Tukey’s multiple comparison test. **p* < 0.05, ***p* < 0.01 and ****p* < 0.001.

**Fig. S6. In vivo biosafety assessment of an oral, sonogenetic system (ultrasound-triggered IL22-EcN/Gel)**

**a** Schematic diagram illustrating the in vivo biosafety assessment of a sonogenetic system. **b and c** H&E staining of histological sections from major organs of healthy mice after oral gavage with IL22-EcN/Gel combined with ultrasound irradiation within one month (3, 7 and 30 days). **d and e** Complete blood count (CBC) analysis of healthy mice after oral gavage with IL22-EcN/Gel combined with ultrasound irradiation within one month (3, 7 and 30days). Data are presented as means ± SD (n=3 biologically independent samples). **f, g and h** Hepatic and renal function analysis of healthy mice after oral gavage with IL22-EcN/Gel combined with ultrasound irradiation within one month (3, 7 and 30 days). Data are presented as means ± SD (n=3 biologically independent samples). P values for **d-h** were calculated by one-way ANOVA followed by Tukey’s multiple comparison test. *p* > 0.05 (not significant, n.s.)

**Fig. S7. The treatment effects in MASH mice from different groups**

**a** Food intake among different groups of mice during the treatment period. **b and c** Liver weight, spleen weight. **d and e** Hepatic TG and hepatic CHOL. **a-e** Data are presented as means ± SD (n=5 biologically independent samples). P values were calculated by one-way ANOVA followed by Tukey’s multiple comparison test. **p* < 0.05, ***p* < 0.01 and *****p* < 0.0001

**Fig. S8. The therapeutic effect of IL22-EcN/Gel combined with US irradiation against HFHC-induced MASH. (a)** Schematic diagram of treatment regimens for different groups. **(b)** Body weight change curves of mice in different groups during the treatment period. **(c)** Digital photographs of the gross morphology of mice from different treatment groups. **(d)** Liver/body weight ratio of mice in different groups. **(e)** Serum AST. **(f)** Serum ALT. **(g)** Serum TG. **(h)** Serum CHOL. **(i)** Serum Glucose. **(j)** Serum INS. **(k-m)** Hepatic SOD **(k)**, hepatic CAT **(l)** and hepatic MDA **(m)**. **(n-p)** Serum IL-1β **(n)**, IL-6 **(o)** and TNF-α **(p)**. **(q)** NAFLD activity scores (NAS) in different groups (n=3 per group). **(r)** Typical liver morphology images and corresponding H&E, Oil Red O and Masson staining sections. Scale bar: 100 μm. Data in **b, d, e, f, g, h,** **i, j, k, l, m, n, o and p** are presented as means ± SD from n = 5 biological independent samples. P values in **b** were calculated by two-way ANOVA. P values in **d-q** were calculated by one-way ANOVA followed by Tukey’s multiple comparison test. **p* < 0.05, ****p* < 0.001 and *****p* < 0.0001.

**Fig. S9. Changes in the relative abundance of Firmicutes and Bacteroidota at the phylum level across different treatment groups in 16S rRNA gene sequencing**

**a** Relative abundance of phylum level gut microbiota among different groups (NCD, CDHFD and Treated **[**IL22-EcN/Gel + US treatment]). **b, c and d** Relative abundance of Firmicutes **(b)**, Bacteroidota **(c)** and Firmicutes/Bacteroidota (F/B) ratio **(d)** at the phylum level among different groups (NCD, CDHFD and Treated **[**IL22-EcN/Gel + US treatment]). Data in **b, c and d** are presented as means ± SD (n=5 biologically independent samples). P values were calculated by two-tailed unpaired student’s t-test. *p* > 0.05 was indicated n.s.

**Fig. S10. Heatmap of the relative abundance of differential species in different treatment groups at the genus and family levels.**

**a** Heatmap of the relative abundance of differential species at the genus level among different groups (NCD, CDHFD and Treated **[**IL22-EcN/Gel + US treatment]). **b** Heatmap of the relative abundance of differential species at the family level among different groups (NCD, CDHFD and Treated **[**IL22-EcN/Gel + US treatment]).

**Fig. S11. Alterations in the fecal metabolomic profile after IL22-EcN/Gel + US treatment**

**a** Different metabolomic profiles of mice from different groups visualized by partial least squares discriminant analysis (PLS-DA). **b** Heatmap of treatment-reversal metabolites across different groups in fecal metabolomics analysis. **c** KEGG pathway analysis of treatment-reversal metabolites. **d** Venn diagram of differential metabolites in fecal metabolomics analysis in NCD group versus CDHFD group and CDHFD group versus treated group (IL22-EcN/Gel + US treatment). **e and f** Volcano plot of differential metabolite expression in NCD group versus CDHFD group **(e)**, CDHFD group versus treated group (IL22-EcN/Gel + US treatment) **(f)**. **g and h** KEGG pathway analysis of differential metabolites between the NCD group versus the CDHFD group **(g)**, the CDHFD group versus the treated group **(h)**. P values were calculated by Fisher’s exact test. ***p* < 0.01, ****p* < 0.001 and *****p* < 0.0001.

**Fig. S12. In vitro gut-liver axis co‑culture model for evaluating the anti‑inflammatory effect of IL22‑EcN + US treatment**

**a** Schematic diagram of the Transwell co-culture system used to simulate the gut-liver axis in vitro. **b** IL-22 expression under different treatments in a Transwell co-culture system measured by ELISA (n=4 biologically independent samples). **c-e** Levels of IL-6 **(c)**, IL-1β **(d)** and TNF-α **(e)** under different treatment groups quantified by ELISA (n=4 biologically independent samples). Data in **b-e** are presented as means ± SD. P values for **b-e** were calculated by one-way ANOVA followed by Tukey’s multiple comparison test. *****p* < 0.0001.

**Fig. S13.** **Heatmap and KEGG pathway enrichment of treatment‑reversal metabolites identified by intersection strategy**

**a** Heatmap of treatment-reversal metabolites across different groups in serum metabolomics analysis. **b** KEGG pathway analysis of treatment-reversal metabolites.

**Fig. S14. Behavioral changes of mice from different treatment groups.**

**a** The latency to platform of different groups of mice during the first 4 days of training in the Morris water maze (MWM) test (n=10 biologically independent experiments). **b** The time in target quadrant of different groups of mice in the MWM test (n=10 biologically independent experiments). **c** The crossing number through target quadrant of different groups of mice in the MWM test (n=10 biologically independent experiments). **d** The preference index of different groups of mice in the novel object recognition (NOR) test (n=10 biologically independent experiments). Data in **a-d** are presented as means ± SD. P values were calculated by one-way ANOVA followed by Tukey’s multiple comparison test. **p* < 0.05, ***p* < 0.01, ****p* < 0.001.

**Fig. S15. Conventional B-mode ultrasonography of liver in different groups of mice (NCD, CDHFD and Treated [IL22-EcN/Gel + US treatment]).**

**Fig. S16.** **Images of H&E, Oil Red O and Masson staining of liver tissues in different groups of mice (NCD, CDHFD and Treated [IL22-EcN/Gel + US treatment]).**

**Fig. S17. Behavioral changes in MASH-associated cognitive impairment rats following IL22-EcN/Gel + US treatment.**

**a** The latency to platform of different groups of rats during the first 4 days of training in the Morris water maze (MWM) test (n=6 biologically independent experiments). **b** Representative swimming trajectories and time heatmaps of different groups of rats in the MWM test. **c** The time in target quadrant of different groups of rats in the MWM test (n=6 biologically independent experiments). **d** The crossing number through target quadrant of different groups of rats in the MWM test (n=6 biologically independent experiments). **e** The crossing number through platform of different groups of rats in the MWM test (n=6 biologically independent experiments). **f** The escape latency of different groups of rats in the MWM test (n=6 biologically independent experiments).

Data in **a, c-f** are presented as means ± SD. P values were calculated by one-way ANOVA followed by Tukey’s multiple comparison test. **p* < 0.05, ***p* < 0.01, ****p* < 0.001.

**Fig. S18. Schematic overview of animal allocation and downstream use of biological samples following behavioral assessments.**

**Supplementary Table 1** Strains used in the study

| **Strains** | **Reference** |
| --- | --- |
| *Escherichia coli* DH5α | Mingzhoubio |
| *Escherichia coli* Nissle 1917 | Mingzhoubio |
| *Escherichia coli* Nissle 1917attλ: lux | Biosci |
| Lux-EcN | this study |
| IL22-EcN | this study |
| IL22-EcN-Lux | this study |

**Supplementary Table 2** Plasmids used in the study

| Plasmids | **Reference** |
| --- | --- |
| pET28a（+） | Unibio |
| pUC57-kan-pSH-IL-22 | Genescript biotech |
| pET28a（+）-pHsh-IL22 | this study |
| ilux pGEX (-) | Addgene |
| pET28a（+）-pHsh-Lux | this study |

**Supplementary Table 3** Primers used in the study

| Primer | Sequence (5’-3’) |
| --- | --- |
| 28A-HSH.F | CTGAAAGGAGGAACTATATCCGGAT |
| 28A-HSH.R | GCGCAACGCAATTAATGTAAG |
| HSH-28A.F | ATCCGGATATAGTTCCTCCTTTCAGttgaatgtgggggaaacatcccca |
| HSH-28A.R | CTAACTTACATTAATTGCGTTGCGCgccgcttcctttcggaagc |
| IL22-CHEKC.F | GACTGGGTTGAAGGCTCTCAA |
| IL22-CHEKC.R | GGCCCACTACGTGAACCATCA |
| 28PSH-LUX.R | GGATCCGCGACCCATTTGCTGTCC |
| 28PSH-LUX.F | TCGACAAGCTTGCGGCCGCACT |
| 1LXC-28PSH.F | GGACAGCAAATGGGTCGCggatccatgactaaaaaaatttcattcattat |
| 1LXC-LXA.R | ggtatgtaagtaaaaagtttccaaatttcatgaattcagagagtccttatattgc |
| 2LXA-LXE.F | gcaatataaggactctctgaattcatgaaatttggaaactttttacttacatacc |
| 2LXA-LXE.R | cttgtttatcaacatatgaagtcatacctgttttactcctcaagataatattagaaag |
| 3LXE-28PSH.R | gccgcaagcttgtcgactcaactatcaaacgcttcggTT |
| 3LXE-LXA.F | ctaatattatcttgaggagtaaaacaggtatgacttcatatgttgataaacaagaa |

**Supplementary Table 4** Primer sequences used for qRT-PCR

| Gene name | Direction | Sequence |
| --- | --- | --- |
| COL1A1 | Forward | CTCCCAGAACATCACCTATCACT |
|  | Reverse | GGAGGTCTTGGTGGTTTTGTATT |
| α-SMA | Forward | GTACCACCATGTACCCAGGC |
|  | Reverse | GAAGGTAGACAGCGAAGCCA |
| GAPDH | Forward | CCTCGTCCCGTAGACAAAATG |
|  | Reverse | TGAGGTCAATGAAGGGGTCGT |
